# On the complexity of non-binary tree reconciliation with endosymbiotic gene transfer

**DOI:** 10.1186/s13015-023-00231-5

**Published:** 2023-07-30

**Authors:** Mathieu Gascon, Nadia El-Mabrouk

**Affiliations:** Département d’Informatique et de Recherche Opérationnelle, Université de Montréal, Montréal, Canada

**Keywords:** Reconciliation, Duplication, Endosymbiotic gene transfer, Multifurcated gene tree, Polytomy

## Abstract

Reconciling a non-binary gene tree with a binary species tree can be done efficiently in the absence of horizontal gene transfers, but becomes NP-hard in the presence of gene transfers. Here, we focus on the special case of *endosymbiotic gene transfers* (EGT), i.e. transfers between the mitochondrial and nuclear genome of the same species. More precisely, given a multifurcated (non-binary) gene tree with leaves labeled 0 or 1 depending on whether the corresponding genes belong to the mitochondrial or nuclear genome of the corresponding species, we investigate the problem of inferring a most parsimonious Duplication, Loss and EGT (DLE) Reconciliation of any binary refinement of the tree. We present a general two-steps method: ignoring the 0–1 labeling of leaves, output a binary resolution minimizing the Duplication and Loss (DL) Reconciliation and then, for such resolution, assign a known number of 0s and 1s to the leaves in a way minimizing EGT events. While the first step corresponds to the well studied non-binary DL-Reconciliation problem, the complexity of the label assignment problem corresponding to the second step is unknown. We show that this problem is NP-complete, even when the tree is restricted to a single polytomy, and even if transfers can occur in only one direction. We present a general algorithm solving each polytomy separately, which is shown optimal for a unitary cost of operation, and a polynomial-time algorithm for solving a polytomy in the special case where genes are specific to a single genome (mitochondrial or nuclear) in all but one species. This work represents the first algorithmic study for reconciliation with endosymbiotic gene transfers in the case of a multifurcated gene tree.

## Introduction

Reconciliation is the process of embedding a gene family tree into a species tree (i.e. reconstructing a mapping between the gene tree and the species tree) to explain how the gene family evolved inside the species tree according to the gene tree model, through evolutionary events modifying gene contents in genomes, such as losses, duplications or horizontal gene transfers (HGTs). This allows deciphering the orthology (divergence through speciation), paralogy (divergence through duplication) or xenology (divergence through HGT) relation between genes, which has important implications on understanding functional specificity of gene copies. For this purpose, the most critical part is the construction of a “good” gene tree, i.e. a gene tree reflecting the true evolution of the nucleotide or amino acid sequences of genes. In fact, as shown in many studies [1], the result of a reconciliation model strongly depends on the considered trees. For example, due to potential errors in the trees, some of the plant datasets analysed in [2] produced unrealistic evolutionary histories with unexpected high number of gene duplications and losses.

**Fig. 1:**
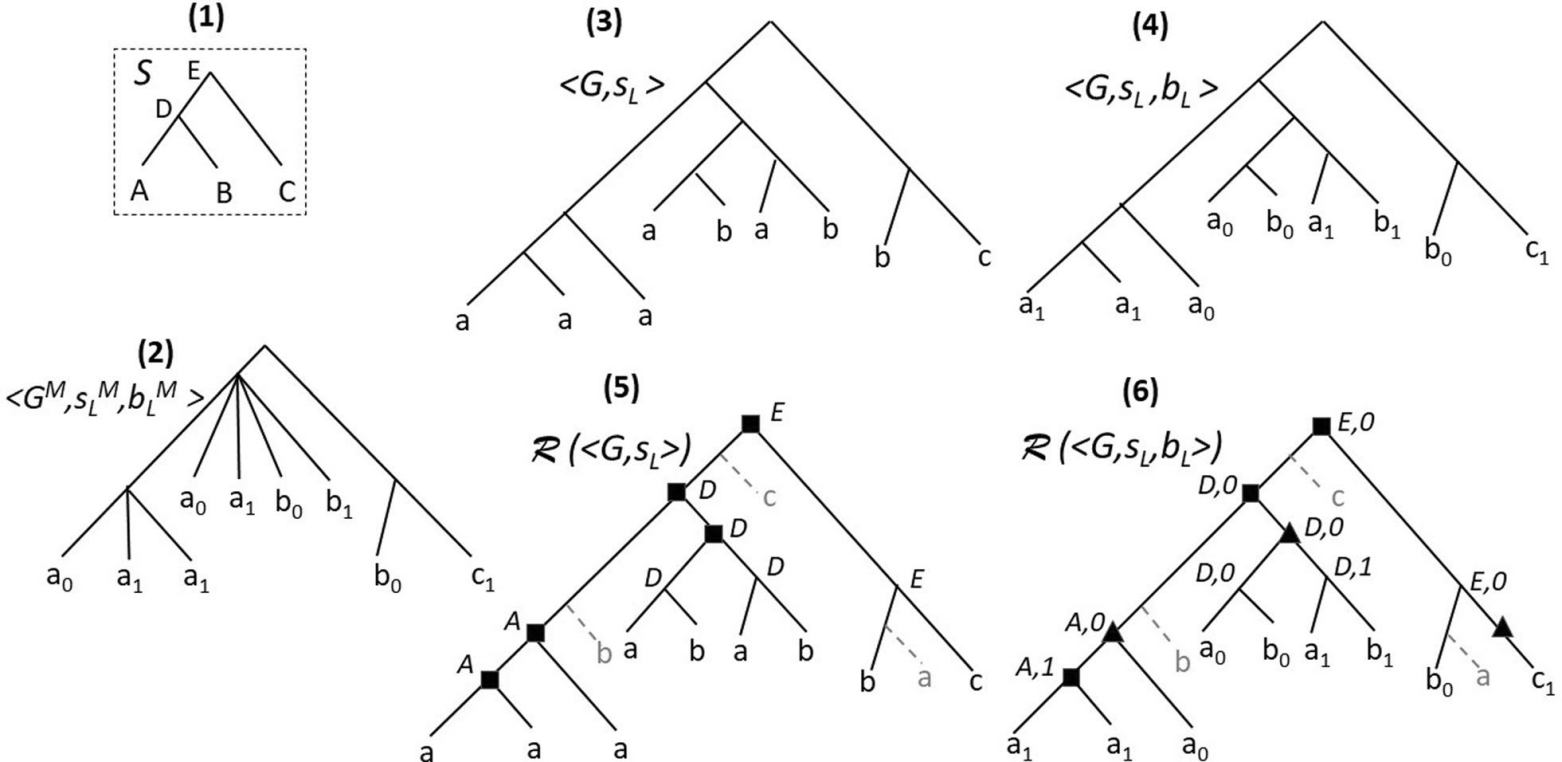
(**1**) A species tree *S* on $\Sigma = \{A,B,C\}$; (**2**) A multifurcated gene tree $G^M$ where leaves are identified by a species mapping $s_L^M$ (a lowercase letter corresponds to the genome identified by the same uppercase letter) and a *b*-mapping $b_L^M$ (the 0–1 index of each leaf); (**3**) a $\langle G,s_L\rangle$ binary refinement of $\langle G^M,s_L^M\rangle$ (i.e. $\langle G^M,s_L^M,b_L^M\rangle$ ignoring the *b*-labeling) and (**4**) a $\langle G,s_L,b_L\rangle$ binary refinement of $\langle G^M,s_L^M,b_L^M\rangle$; (**5**) A DL-Reconciliation of $\langle G,s_L\rangle$ and (**6**) a DLE-Reconciliation of $\langle G,s_L,b_L\rangle$. The internal node labeling corresponds to the LCA-mapping with *S*, squares correspond to duplications, triangles to EGTs, dotted lines to losses and unary nodes to EGTLs. The *s* and *b*-labeling of nodes with a lost child are omitted. For a unitary cost of operations, the DLE-Reconciliation is of cost 9. It is optimal for the DLE-BinL problem

**Fig. 2:**
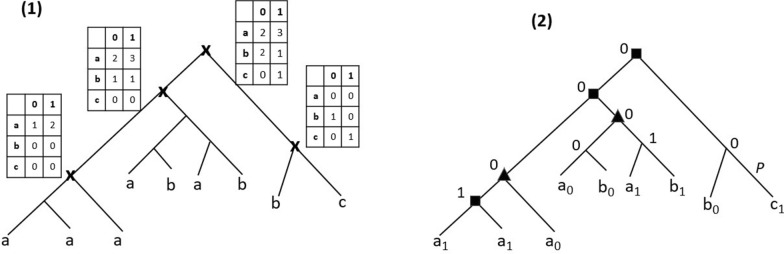
(**1**) A binary refinement $\langle G,s_L\rangle$ of the multifurcated tree of Fig. 1.(2) and the corresponding *b*-constraint labeling (*M*, *I*): *I* is the set of nodes indicated by crosses, and for each such node *x*, *M*(*x*) is the table represented at that node; (**2**) The $b_L$ assignment leading to the optimal DLE-Reconciliation, also represented in Fig. 1.(6). Here, the compressed DLE-Reconciliation is illustrated, where the edge labeled *P* is the only one where an EGTL event is present

Unfortunately, for many reasons related to sequence alignment, limitations of the considered phylogenetic method or issues with the sequence dataset (not enough mutations or too many, both cases leading to absence of signal), gene trees are almost never inferred with absolute certainty. As phylogenetic reconstruction methods are usually accompanied with statistical evaluations on branches, a solution for removing ambiguities in a tree is collapsing its weakly supported branches, leading to a non-binary tree (tree with multifurcated nodes, also called polytomies). The problem then becomes one of simultaneously finding a binary refinement and optimal reconciliation of the multifurcated tree, more precisely, inferring an optimal evolutionary scenario leading to a binary refinement of the tree. This strategy has been applied, for example, to infer the evolution of the gene families responsible for alkaloid accumulation in plants [3].

Reconciling a non-binary gene tree with a binary species tree can be done efficiently in the absence of HGTs (a review can be found in [4]). As far as we know, the most efficient algorithm for minimizing a Duplication/Losses (DL) distance is PolytomySolver [5], which handles unit costs in linear time, improves the best complexity of previous algorithms for the general DL cost model by a linear factor and enables to account for various evolutionary rates across the branches of a species tree. However, the problem becomes NP-hard in the presence of gene transfers [6]. Various heuristics have been developed for the DTL (Duplication, Transfer, Loss) reconciliation of a non-binary gene tree with a binary species tree [7–9].

**Fig. 3:**
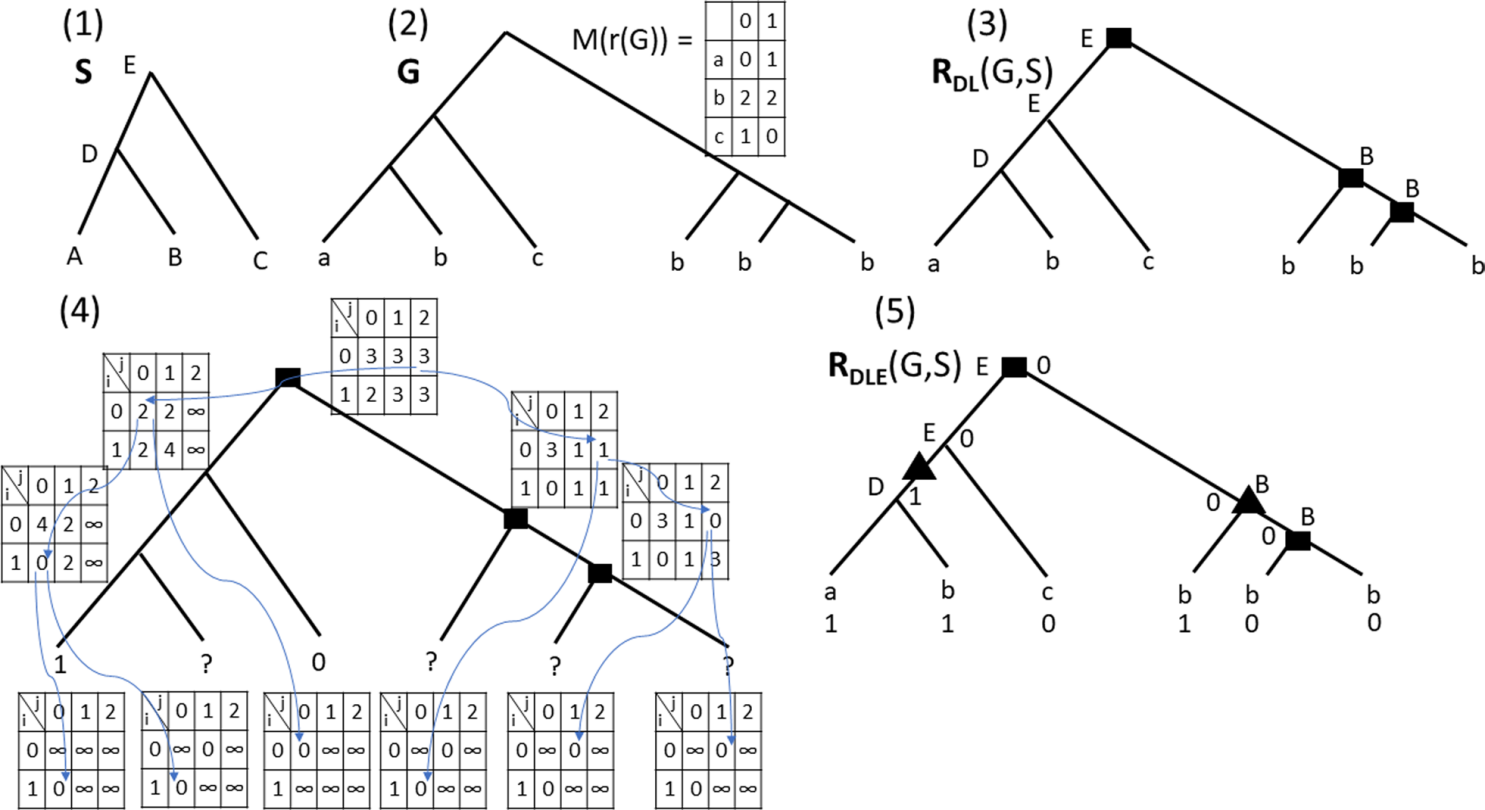
(**1**) A species tree *S* on $\Sigma = \{A,B,C\}$; (**2**) A binary gene tree *G* where leaves are identified by a species mapping *s*, and a b-Constraint (*M*, *I*) where $I= r(G)$; (**3**) An optimal DL-Reconciliation of *G* with *S*; (**4**) The tree *G* accompanied with the arrays computed by Algorithm 6 (we consider here the costs $\delta = \lambda = 1$ and $\rho = \tau = 2$) and the pointers for an optimal solution; (**5**) The optimal DLE-Reconciliation ${\mathcal {R}}_{DLE}(G,S)$ of $\langle G,s_L,b_L\rangle$ (where $b_L$ is consistent with (*M*, *I*)) returned by Algorithm 5. The cost $minCostTransfer({\mathcal {R}}_{DLE}(G,S))$ is 3. Events are represented as in Fig. 1

**Fig. 4:**
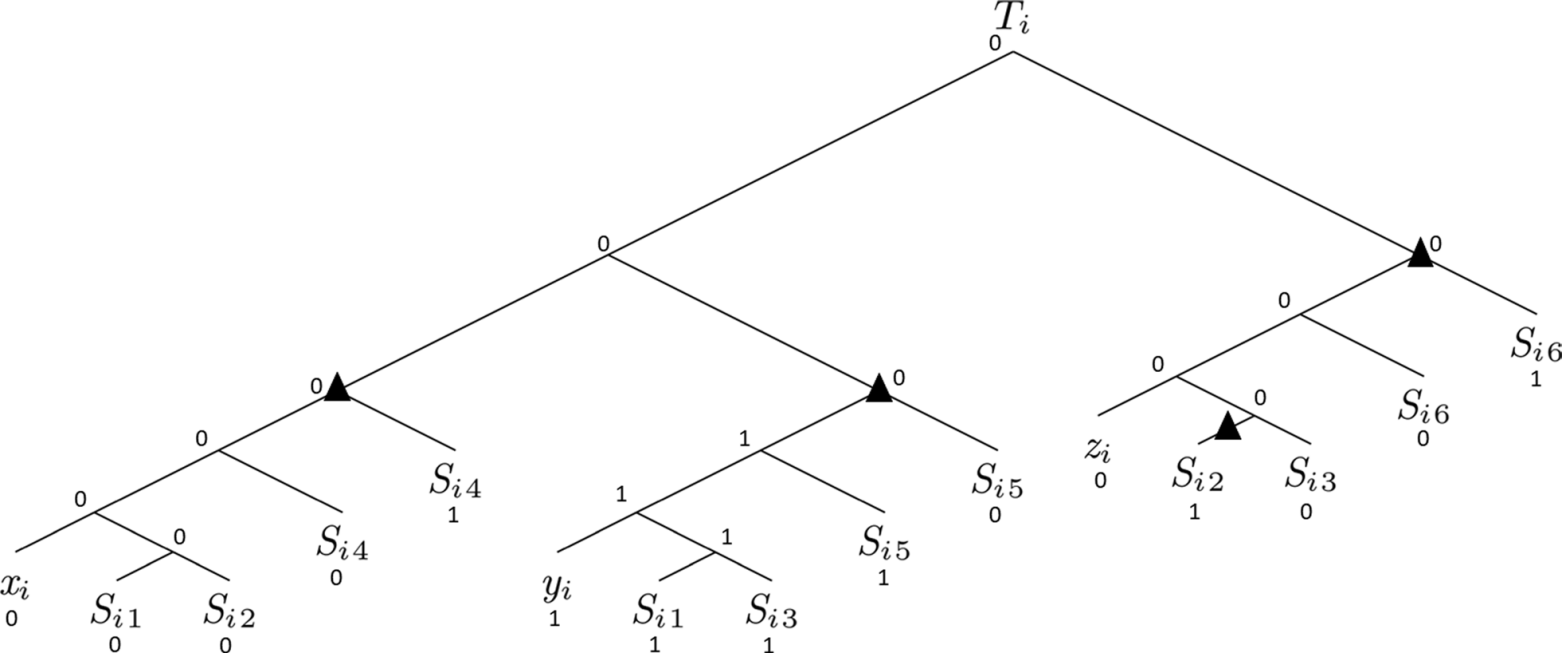
A valid *b*-labeling of $T_i$ requiring one EGTL event

In this paper, we focus on the particular case of DTL non-binary gene tree reconciliation, where transfers can only move genes between the mitochondrial and nuclear genome of the same species – called endosymbiotic gene transfers. In fact, it is well known that episodes of such gene transfers, mainly from the mitochondria to the nucleus, have marked the eukaryote evolution since an initial endosymbiotic event integrating an $\alpha -$proteobacterial genome into an eukaryotic cell, which is known to be at the origin of all extent mitochondria. Such events resulted in a significant reduction of the mitochondrial genome. Understanding how both nuclear and mitochondrial genomes have been shaped by gene loss, duplication and transfer is important to shed light on a number of open questions regarding the origin, evolution, and characteristics of gene coding capacity of eukaryotes, but also on the rooting of the eukaryotic tree.

From a computational point of view, EndoRex [2] is the first algorithm developed for integrating such endosymbiotic events in a reconciliation model. Given a gene family with gene copies labeled by 0 or 1 depending on whether they are encoded in the mitochondrial or nuclear genome of a given species, a binary gene tree for the gene family and a binary species tree for the considered species, EndoRex infers a most parsimonious scenario of duplications, losses and endosymbiotic gene transfers (EGT) explaining the gene tree given the species tree. It is an exact polynomial-time algorithm, which can be used to output all minimum cost solutions, for arbitrary costs of operations.

Here, we explore the case of a non-binary gene tree. More precisely, given a multifurcated gene tree for a gene family with 0–1 labeled genes (leaflabels of the gene tree), the problem consists in inferring a most parsimonious duplication, loss and EGT scenario leading to a binary refinement of the tree. Our method is in two steps: ignoring the 0–1 labeling of the gene tree leaves, output all resolutions minimizing the DL-Reconciliation cost and then, for each resolution (i.e. binary tree), assign a known number of 0s and 1s to the leaves in a way minimizing EGT events. Step one can be done efficiently as recalled above. Therefore, we focus on the second step which consists in assigning a 0–1 labeling to the nodes of a binary tree, in a way minimizing the considered evolutionary distance. We show in "Complexity of the dle-binl and dle-binl1 Problems" and "The one-direction DLE-reconciliation problem" sections that this problem is NP-complete, even when the tree is restricted to a single multifurcated node (also called polytomy) and, surprisingly, even if transfers can occur in a single direction (e.g. from the mitochondrial to the nuclear genome). It is polynomial in the very restricted case of a binary tree obtained as an optimal refinement (step 1) of a star-tree, and with each leaflabel present at most a fixed number of times. We then, in "A general algorithm for the dle-binl problem" section, present a general algorithm solving each polytomy separately, which is shown optimal for a unitary cost of operations.

Except for species conserving the traces of an ancestral eukaryotic origin, few genes are expected to reflect an intermediate endosymbiotic integration of the mitochondrial gene content to the nucleus, with gene copies in both the nuclear and mitochondrial genome. This is the case of the eukaryotes with complete mitochondrial genomes explored in [10] (statistics summarized in [2]): among the 2,486 species, only 52 species have mitochondrial-encoded genes also present in the nuclear genome. This motivates "An exact algorithm for the one-species version of the dle-binl1 problem" where we develop a polynomial-time algorithm for the *b*-labeling problem in the special case where, in each polytomy, genes are specific to a single genome (mitochondrial or nuclear) in all but one species. We first begin, in the next section, by formally defining our problems.

## Preliminaries, evolutionary model and definitions

All trees are considered rooted. Given a tree *T*, we denote by *r*(*T*) its root, by *V*(*T*) its set of nodes and by $L(T) \subseteq V(T)$ its leafset. We call $n=|L(T)|$ the *size* of *T*. A node *x* is a *descendant* of *y* if *x* is on the path from *y* to a leaf of *T* and an *ancestor* of *y* if *x* is on the path from *r*(*T*) to *y*; *x* is a *strict descendant* (respect. *strict ancestor*) of $x'$ if it is a descendant (respec. ancestor) of $x'$ different from $x'$. Moreover, *x* is the *parent* of $y\ne r(T)$, denoted *p*(*y*), if it directly precedes *y* on this path. In this latter case, *y* is a *child* of *x*. We denote by *E*(*T*) the set of edges of *T*, where an edge is represented by its two terminal nodes (*x*, *y*), with *x* being the parent of *y*. More generally, if *x* is an ancestor of *y*, (*x*, *y*) denotes the path between *x* and *y*. The subtree of *T* rooted at *x* (i.e. containing all the nodes descendant from *x* in *T*) is denoted *T*[*x*]. The *lowest common ancestor* (LCA) in *T* of a subset $L'$ of *L*(*T*), denoted $lca_T(L')$, is the ancestor common to all the nodes in $L'$ which is the most distant from the root.

An internal node (a node which is not a leaf) is said to be *unary* if it has a single child, *binary* if it has two children, and a *polytomy* if it has at least two children. Moreover, a *star-tree* is a tree with a single internal node. We will denote by $x_l$ and $x_r$ the two children of a binary node. The node $x_l$ (respec. $x_r$) is called *the sibling* of $x_r$ (respec. $x_l$).

A tree *R* is *an extension* of a tree *T* if it is obtained from *T* by *grafting* unary or binary nodes in *T*, where grafting a unary node *x* on an edge (*u*, *v*) consists in creating a new node *x*, removing the edge (*u*, *v*) and creating two edges (*u*, *x*) and (*x*, *v*), and in the case of grafting a binary node, also creating a new leaf *y* and an edge (*x*, *y*). In the latter case, we say that *y* is a *grafted leaf*. Moreover, given a function *f* defined from *U* to *V*, *an extension* $f'$ of *f* is a function defined from $U'$ to $V'$ with $U\subset U'$ and $V\subseteq V'$ such that for any $x\in U$, $f'(x) = f(x)$.

A *species tree* for a set ${\Sigma }$ of species is a tree *S* with a bijection between *L*(*S*) and ${\Sigma }$. In this paper, we assume that the species tree *S* for a given set of species ${\Sigma }$ is known, rooted and binary. For example, the tree *S* in Fig. 1.(1) is a species tree for the set of species ${\Sigma }= \{A,B,C\}$. A *gene family* is a set ${\Gamma }$ of genes where each gene $x \in {\Gamma }$ belongs to a given species $s_L(x)$ of ${\Sigma }$. A tree *G* is a *gene tree* for a gene family ${\Gamma }$ if its leafset is in bijection with ${\Gamma }$. We write $\langle G,s_L\rangle$ when each leaf of *G* is meant to be fully identified by its *species labeling*, i.e. the species $s_L(x)$ it belongs to (e.g. gene tree in Fig. 1.(3); lowercase letters represent genes in the genome represented by the same letter in uppercase).

In this paper, we will consider an additional *b*-*labeling* for a gene *x*: $b_L(x)= 0$ if *x* belongs to the mitochondrial genome of $s_L(x)$, and $b_L(x)= 1$ if *x* belongs to the nuclear genome of $s_L(x)$. We write $\langle G,s_L,b_L\rangle$ when we want to specify that each leaf of *G* is fully identified by these two labels (e.g. trees (2) and (4) in Fig. 1). To summarize, *G*, $\langle G,s_L\rangle$ and $\langle G,s_L,b_L\rangle$ are three notations for a gene tree, the two last specifying the way the leaves of *G* are identified. Later, we will need to define labeling for internal nodes of *G*.

A *binary tree* is a tree with all internal nodes being binary. If internal nodes have one or two children, then the tree is said *partially binary*. A *multifurcated tree* is a tree containing at least one polytomy. For example, in Fig. 1, the tree (2) is a multifurcated tree with two polytomies.

### Definition 1

(binary refinement) Let $\langle G^M,s_L^M,b^M_L\rangle$ be a multifurcated tree. A binary tree $\langle G,s_L,b_L\rangle$ is said to be a *binary refinement* of $\langle G^M,s^M_L,b^M_L\rangle$ if $V(G^M) \subseteq V(G)$ and for every $x \in V(G^M)$, $L(\langle {G}^M,{s^M}_L,{b^M}_L\rangle [x]) = L(\langle G,{s_L},{b_L}\rangle [x])$. We denote by ${\mathcal {B}}(\langle G^M,s^M_L,b^M_L\rangle$ the set of binary refinements of $\langle G^M,s^M_L,b^M_L\rangle$.

As for a multifurcated tree $\langle G^M,s^M_L\rangle$, a binary refinement $\langle G,s_L\rangle$ and the set of binary refinements ${\mathcal {B}}(\langle G^M,s^M_L\rangle )$ are defined in the same way, just ignoring the *b*-labeling.

In Fig. 1, the tree in (4) is a binary refinement of the tree in (2), and the tree in (3) is the same binary refinement, just ignoring the 0–1 labeling of leaves.

We need a final notation. Let $X\subseteq L(\langle G,s_L,b_L\rangle )$. The *count matrix* *Count*(*X*) for *X* is a $|\Sigma | \times 2$ matrix defined as follows:

$$\begin{aligned} {\left\{ \begin{array}{ll} Count(X)[\sigma ,0] &{}= \hbox {number of genes } g \in X \hbox { such that } s_L(g) = \sigma \hbox { and } b_L(g) = 0\\ Count(X)[\sigma ,1] &{}= \text {number of genes } g \in X \hbox { such that } s_L(g) = \sigma \hbox { and } b_L(g) = 1 \end{array}\right. } \end{aligned}$$

### DLE reconciliation

Inside the species’ genomes, genes undergo *Speciation* (Spe) when the species to which they belong do, but also *Duplication* (Dup) i.e. the creation of a new gene copy, *Loss* of a gene copy, and transfer when a gene is transmitted from a source to a target genome. In this paper, we only consider endosymbiotic gene transfers, denoted *EGT*, i.e. the special case of transfers only allowing the transmission of genes from the mitochondrial genome to the nuclear genome of the same species, or vice-versa. If the transmission of a gene from a genome *A* to a genome *B* is accompanied by the loss of the gene in *A*, we refer to the event as an *EGTL* for ($EGT-Loss$) event.

We are now ready to recall the definition of a DLE-Reconciliation as introduced in [2].

#### Definition 2

(DLE-Reconciliation) Let $\langle G,s_L,b_L\rangle$ be a rooted binary gene tree for a gene family $\Gamma$ and *S* be a rooted binary species tree for the species $\Sigma$ the genes belong to. A *DLE-Reconciliation* of $\langle G,s_L,b_L\rangle$ with *S* (or simply DLE-Reconciliation if no ambiguity) is a quadruplet $\langle R,s,b,e\rangle$ where *R* is a partially binary extension of *G*, *s* is an extension of $s_L$ from *V*(*R*) to *V*(*S*), *b* is an extension of $b_L$ from *V*(*R*) to $\{0,1\}$, and *e* is an event labeling of the internal nodes of *R*, such that:

1. Each unary node *x* with a single child *y* is such that $e(x)=EGTL$, $s(x) = s(y)$ and $b(x) \ne b(y)$; *x* is an EGTL event with source genome $\sigma _{b(x)}$ and target genome $\sigma _{b(y)}$, where $\sigma =s(x)$ (or equivalently *s*(*y*)).
2. For each binary node *x* of *R* with two children $x_l$ and $x_r$, one of the following cases holds:
  - $s(x_l)$ and $s(x_r)$ are the two children of *s*(*x*) in *S* and $b(x_l)= b(x_r)=b(x)$, in which case $e(x)=Spe$;
  - $s(x_l) = s(x_r) = s(x) = \sigma$ and $b(x_l)= b(x_r)=b(x)$ in which case $e(x)=Dup$ representing a duplication in $\sigma _{b(x)}$;
  - $s(x_l) = s(x_r) = s(x) = \sigma$ and $b(x_l)\ne b(x_r)$ in which case $e(x)=EGT$; let *y* be the element of $\{x_l,x_r\}$ verifying $b(x) \ne b(y)$, then *e*(*x*) is an EGT with source genome $\sigma _{b(x)}$ and target genome $\sigma _{b(y)}$.

Grafted leaves in the extension *R* correspond to gene losses.

As *R* is as an extension of *G*, each node in *G* has a corresponding node in *R*. In particular, the *s*, *b* and *e* labeling on *R* induce an *s*, *b* and *e* labeling on the nodes of *G*. The difference between *G* and *R* are additional binary nodes with a child being a grafted leaf (a loss), and unary nodes corresponding to EGTL events.

A *DL-reconciliation* of $\langle G,s_L\rangle$ is defined as in Definition 2, ignoring the *b*-labeling, i.e. it is a tuple $\langle R,s,e\rangle$ where *R* is an extension of *G*. For example, in Fig. 1, (5) is a DL-Reconciliation of the gene tree in (3), and (6) is a DLE-Reconciliation of the tree in (4).

*Optimal reconciliation:* Let *c* be a function attributing a cost to each event in $DLE= \{Spe, Dup, Loss, EGT, EGTL\}$. As it is usually the case, we will assume a 0 cost for speciations and positive costs for all the other events. Moreover, we assume that $c(Dup) < c(EGT)+c(EGTL)$ as otherwise duplications could be never inferred in a most parsimonious reconciliation. Similarly, we assume $c(EGT) < c(Dup) +c(EGTL)$ to allow for EGTs and $c(EGTL) < c(EGT) +c(Loss)$ to allow for EGTLs.

Given a DLE-Reconciliation ${\mathcal {R}}=\langle R,s,b,e\rangle$ (respec. DL-Reconciliation $\langle R,s,e\rangle$), the cost $C({\mathcal {R}})$ of ${\mathcal {R}}$ is the sum of costs of the events labeling the internal nodes of *R* plus the sum of costs of the losses, i.e. $C({\mathcal {R}})= \sum _{x\in V(R) {\setminus } L(R)}c(e(x)) + |L(R)_{Loss}| * c(Loss)$ where $|L(R)_{Loss}|$ is the number of losses in ${\mathcal {R}}$. In this paper, we seek for a most parsimonious reconciliation, i.e. a reconciliation of minimum cost, also called *optimal reconciliation*. We denote by *DLE*(*G*, *S*) (respec. *DL*(*G*, *S*)) the cost of an optimal DLE-Reconciliation (respec. DL-Reconciliation).

From now on, we denote by $\delta$, $\lambda$, $\tau$ and $\rho$ respectively, the cost of a duplication, a loss, an EGT and an EGTL event. The cost function is said to be *unitary* when $\delta = \lambda = \tau = \rho$.

The following lemma makes the link between an optimal DLE-Reconciliation and the optimal DL-Reconciliation.

#### Lemma 1

Any optimal DLE-Reconciliation ${\mathcal {R}}_{DLE}=\langle R_{DLE},s_{DLE},b_{DLE},e_{DLE}\rangle$ of $\langle G,s_L,b_L\rangle$ can be obtained from the optimal DL-Reconciliation ${\mathcal {R}}_{DL}=\langle R_{DL},s_{DL},e_{DL}\rangle$ where ${\mathcal {R}}_{DLE}$ is obtained from ${\mathcal {R}}_{DL}$ by possibly adding unary nodes (corresponding to EGTLs), $s_{DLE}$ is an extension of $s_{DL}$ and $e_{DLE}$ is obtained from $e_{DL}$ by labeling unary nodes as EGTLs and possibly converting duplications into EGTs.

#### Proof

Let’s consider, by contradiction, an optimal DLE-Reconciliation ${\mathcal {R}}_{DLE}$ of $\langle G,s_L,b_L\rangle$ that cannot be obtained from the optimal DL-Reconciliation by possibly adding unary nodes and possibly converting duplications into EGTs. Let’s now consider the DL-Reconciliation ${\mathcal {R}}_{DL}$ obtained from ${\mathcal {R}}_{DLE}$ by removing all unary nodes, converting all EGTs into duplications and ignoring the binary assignement of genes. Let *x* be a duplication of ${\mathcal {R}}_{DL}$ with at least one loss as a child. By construction of ${\mathcal {R}}_{DL}$, *x* is either a duplication or an EGT node in ${\mathcal {R}}_{DLE}$.

1. If *x* is a duplication in ${\mathcal {R}}_{DLE}$, then removing this duplication and one of its loss child and connecting its other child to its parent (if the *x* is the root then its other child becomes the new root) would result in a DLE-Reconciliation ${\mathcal {R}}'_{DLE}$ which cost is lower than $C({\mathcal {R}}_{DLE})$. This contradicts the fact that ${\mathcal {R}}_{DLE}$ is optimal.
2. If *x* is an EGT in ${\mathcal {R}}_{DLE}$, then replacing this EGT by an EGTL node and removing its loss child from ${\mathcal {R}}_{DLE}$ would result in a DLE-Reconciliation ${\mathcal {R}}'_{DLE}$ which cost is lower than $C({\mathcal {R}}_{DLE})$ (because we assume $c(EGT)+c(Loss) > c(EGTL$)). This also contradicts the fact that ${\mathcal {R}}_{DLE}$ is optimal.

Therefore, ${\mathcal {R}}_{DL}$ has no duplication node with a loss as a child and thus all duplication nodes of ${\mathcal {R}}_{DL}$ have a corresponding node in *G*. Let ${\mathcal {R}}^*_{DL}$ be the optimal DL-Reconciliation of *G* with *S*. Note that ${\mathcal {R}}_{DL}$ cannot have less duplication nodes than ${\mathcal {R}}^*_{DL}$ as the optimal DL-Reconciliation has the minimum number of duplication nodes possible for a DL-Reconciliation [11]. As each duplication node in ${\mathcal {R}}_{DL}$ has a corresponding node in *G*, it has also a corresponding node in ${\mathcal {R}}^*_{DL}$. If each such duplication node in ${\mathcal {R}}_{DL}$ is also a duplication node in ${\mathcal {R}}^*_{DL}$, then ${\mathcal {R}}_{DL}={\mathcal {R}}^*_{DL}$, which is in contradiction with the hypothesis. Therefore, there is a least one duplication node *x* in ${\mathcal {R}}_{DL}$ which corresponding node in ${\mathcal {R}}^*_{DL}$ is a speciation. Both the children of *x* in ${\mathcal {R}}_{DL}$ must have a loss as a child as otherwise *x* would be a speciation. Similarly to the previous case, *x* is either a duplication or an EGT in ${\mathcal {R}}_{DLE}$ and removing the loss children of its two children (and eventually adding an EGTL event if needed) results in a DLE-Reconciliation ${\mathcal {R}}'_{DLE}$ with *x* transformed into a speciation, and thus $C({\mathcal {R}}'_{DLE})<C({\mathcal {R}}_{DLE})$. This is a contradiction as we supposed ${\mathcal {R}}_{DLE}$ to be optimal. $\square$

Recall that the optimal DL-Reconciliation is unique and $s_{DL}$ is the LCA-mapping [4], i.e. for each node *x* of ${\mathcal {R}}_{DL}$ corresponding to a node of *G*, $s_{DL}(x)=lca_S(\{s_L(g): g \in G[x]\})$. Moreover, as $s_{DLE}$ is an extension of $s_{DL}$ and ${\mathcal {R}}_{DLE}$ is an extension of ${\mathcal {R}}_{DL}$, for each node *x* of *G*, $s_{DLE}(x) = s_{DL}(x)$. See for an example the optimal DLE-Reconciliation in Fig. 1.(6), obtained from the optimal DL-Reconciliation (5) by converting two duplication nodes into EGT nodes and adding an EGTL unary node on the terminal edge leading to the gene in genome *C*.

Given a DLE-Reconciliation ${\mathcal {R}}_{DLE}$, removing an even number of consecutive EGTL nodes can only lead to a more parsimonious DLE-Reconciliation. Therefore, we assume that a reconciliation does not involve such nodes. This assumption is used in the following definition of a compressed reconciliation.

#### Definition 3

(Compressed reconciliation) A *compressed DLE-Reconciliation* of $\langle G,s_L,b_L\rangle$ is a tuple $\langle G,s,b,e_V,e_E\rangle$ obtained from a DLE-Reconciliation $\langle R,s,b,e\rangle$ of $\langle G,s_L,b_L\rangle$, where $e_V$ is simply *e* restricted to the nodes of *G* and $e_E$ is a P/A (Presence/Absence) labeling of the edges of *G* indicating the presence or absence of an EGTL node on that edge, i.e. obtained as follows: Let $G'$ be the tree obtained from *R* by removing grafted leaves and their parental nodes (i.e. ignoring losses). For each edge (*x*, *y*) of *G*, let $x', y'$ be the corresponding nodes in $G'$ ($G'$ differs from *G* only by unary nodes). Then:

$$\begin{aligned} e_E(x,y) = {\left\{ \begin{array}{ll} P &{} \text {if the path } (x',y') \text { in } G' \text { contains a unary node}\\ A &{} \text {if the path } (x',y') \text { in } G' \text { contains no unary node} \end{array}\right. } \end{aligned}$$

A *compressed DL-Reconciliation* of $\langle G,s_L\rangle$ is defined similarly, ignoring *b* and the $e_E$ labeling. For example, in Fig. 1, the compressed DL-Reconciliation of (5) is simply that tree ${{\mathcal {R}}}(\langle G,s_L\rangle )$ where we ignore losses, i.e. dotted lines. Moreover, the compressed DLE-Reconciliation of (6) is that tree $\mathcal{R}(\langle G,s_L,b_L\rangle )$ where we ignore losses and replace the unary node (EGTL) on the branch leading to $c_1$ by a label on that branch.

For a compressed DLE-Reconciliation ${\mathcal {R}}^c =\langle G,s,b,e_V,e_E\rangle$, denote by $|e_{V_{EGT}}|$ the number of EGT nodes, by $|e_E|$ the number of edges labeled *P*, i.e. the number of EGTL events, and define the cost of ${\mathcal {R}}^c$ as $C({\mathcal {R}}^c) = DL(G,S) + |e_{V_{EGT}}|* (\tau - \delta ) + |e_E|* \rho$.

#### Lemma 2

From a *compressed DLE-Reconciliation* ${\mathcal {R}}^c =\langle G,s,b,e_V,e_E\rangle$ for $\langle G,s_L,b_L\rangle$, we can obtain a DLE-Reconciliation ${\mathcal {R}}$ of $\langle G,s_L,b_L\rangle$ of cost $C({\mathcal {R}}) = C({\mathcal {R}}^c)$ in linear time.

#### Proof

Let ${\mathcal {R}}^c =\langle G,s,b,e_V,e_E\rangle$ be a compressed DLE-Reconciliation for $\langle G,s_L,b_L\rangle$.

Let ${\mathcal {R}}_{DL} =\langle R_{DL},s,e_{DL}\rangle$ be the optimal DL-Reconciliation of *G* with *S*. We construct a DLE-Reconciliation ${\mathcal {R}}= \langle R_{DLE},s_{DLE},b_{DLE},e_{DLE}\rangle$ from ${\mathcal {R}}_{DL}$ and ${\mathcal {R}}^c$ in linear time as follows:

- $R_{DLE}$ is obtained from $R_{DL}$ by grafting a unary node (EGTL) on the edge (*p*(*x*), *x*) (in $R_{DL}$) for each node $x \in V(R_{DL}) \cap V(G)$ such that $e_E(p(x),x) = P$.
- $s_{DLE}$ is the LCA-mapping.
- $e_{DLE}(x) = e_{DL}(x)$ for each node $x \in V(R_{DL}) \cap V(R_{DLE})$ and $e_{DLE}(x) = EGTL$ for each unary node of $R_{DLE}$. For each node $x \in V(G) \cap V(R_{DLE})$, if $e_V(x) = EGT$ then we set $e_{DLE}(x) = EGT$.
- $b_{DLE}(x) = b(x)$ for each node $x \in V(R_{DLE}) \cap V(G)$. For each node $x \in V(R_{DLE}) \setminus V(G)$, let *y* be the lowest ancestor of *x* such that $y \in V(R_{DLE}) \cap V(G)$. If *y* is not an EGT node, then set $b_{DLE}(x) = b(y)$ if there is no EGTL event in the path (*y*, *x*) (in $R_{DLE}$), and set $b_{DLE}(x) = 1 - b(y)$ otherwise. Else if *y* is an EGT node, set $b_{DLE}(x) = b(y)$ if the EGT node *y* does not transfer in the direction of *x* and $b_{DLE}(x) = 1 - b(y)$ otherwise.

As ${\mathcal {R}}$ is constructed from ${\mathcal {R}}_{DL}$, it is easy to see that the species labeling of the nodes of $R_{DLE}$ is correct. By construction, the *b*-labeling of the nodes of $R_{DLE}$ is also correct, as the *b*-labeling *b* is assumed correct (thus the *b*-labeling of the nodes $x \in V(R_{DLE}) \cap V(G)$ is correct) and the *b*-labeling of the nodes $x \in V(R_{DLE}) \setminus V(G)$ is set according to the definition.

Notice that there are $|e_E|$ EGTL events and $|e_{V_{EGT}}|$ EGT events in ${\mathcal {R}}$. Also, the number of loss events in ${\mathcal {R}}$ is the same as the number of loss events in ${\mathcal {R}}_{DL}$. Let $|e_{DL_{Dup}}|$ be the number of duplication nodes in the DL-Reconciliation. As an EGT event in ${\mathcal {R}}$ may only occur on a node that is a duplication in ${\mathcal {R}}_{DL}$, there are $|e_{DL_{Dup}}| - |e_{V_{EGT}}|$ duplication events in ${\mathcal {R}}$. Therefore, the cost of ${\mathcal {R}}$ is: $C({\mathcal {R}}) = DL(G,S) + |e_{V_{EGT}}|* (\tau - \delta ) + |e_E|* \rho$ $\square$

#### Corollary 1

From an optimal compressed DLE-Reconciliation ${\mathcal {R}}^c=\langle G,s,b,e_V,e_E\rangle$, an optimal DLE-Reconciliation ${\mathcal {R}}$ of $\langle G,s_L,b_L\rangle$ can be obtained in linear time.

#### Proof

For a compressed DLE-Reconciliation ${\mathcal {R}}^c = \langle G,s,b,e_V,e_E\rangle$, a DLE-Reconciliation leading to ${\mathcal {R}}^c$, of the same cost as ${\mathcal {R}}^c$, can be found in linear-time by the constructive proof of Lemma 2. In particular, a DLE-Reconciliation ${\mathcal {R}}$ can be obtained from an optimal compressed DLE-Reconciliation ${\mathcal {R}}^c$, and this DLE-Reconciliation ${\mathcal {R}}$ is necessarily optimal. In fact, from Lemma 1, any optimal DLE-Reconciliation ${\mathcal {R}}_{DLE}$ can be obtained from the optimal DL-Reconciliation. Then, by construction of ${\mathcal {R}}_{DLE}$, $C({\mathcal {R}}_{DLE}) = DL(G,S) + |e_{V_{EGT}}|* (\tau - \delta ) + |e_E|* \rho$, which is also the cost of its compressed DLE-Reconciliation ${\mathcal {R}}^c_{DLE}$. But as ${\mathcal {R}}^c$ is optimal, $C({\mathcal {R}}^c) \le C({\mathcal {R}}^c_{DLE})$, and thus $C({\mathcal {R}}) \le C({\mathcal {R}}_{DLE})$, but as ${\mathcal {R}}_{DLE}$ is by definition an optimal DLE-Reconciliation, we have $C({\mathcal {R}}) = C({\mathcal {R}}_{DLE})$ and thus ${\mathcal {R}}$ is also optimal. $\square$

The problem of finding an optimal DLE-Reconciliation is thus equivalent to that of finding an optimal compressed DLE-Reconciliation.

By default, we will consider compressed DLE-Reconciliations unless we explicitly state that the considered reconciliation is non-compressed.

### Problem statements

The general problem of simultaneously refining and reconciling a multifurcated gene tree under the DLE evolutionary model is formulated as follows.

DLE Non-binary Reconciliation problem:

**Input:** A binary species tree *S*, a multifurcated gene tree $\langle G^M,s^M_L,b^M_L\rangle$ and a cost function *c* on DLE.

**Output:** An optimal DLE-Reconciliation $\langle G,s,b,e_V, e_E\rangle$ of $\langle G,s_L,b_L\rangle$ over all $\langle G,s_L,b_L\rangle \in {\mathcal {B}}(\langle G^M,s^M_L,b^M_L\rangle )$.

The DL Non-binary Reconciliation problem is simply the restriction of the previous problem to DL-Reconciliation.

The complexity of the DLE Non-binary Reconciliation problem Problem is unknown. Our resolution method for this problem operates in two steps:

**Resolution method:**

**Step 1:** Find a binary refinement $\langle G,s_L\rangle$ of $\langle G^M,s^M_L\rangle$ leading to an optimal DL-Reconciliation.

**Step 2:** Given the binary tree $\langle G,s_L\rangle$ obtained above, find a *b*-labeling $b_L$ such that $\langle G,s_L,b_L\rangle$ is a binary refinement of $\langle G^M,s^M_L,b^M_L\rangle$ leading to an optimal DLE-Reconciliation $\langle G,s,b,e_{V},e_E\rangle$.

Although not guaranteed to be optimal, this method is a natural greedy heuristic for the DLE Non-binary Reconciliation problem. In fact, as stated in Lemma 1, an optimal DLE binary reconciliation (result of Step 2) is obtained from a DL binary reconciliation (result of Step 1) by simply converting some duplication nodes into EGT nodes and adding EGTL labels on branches. Moreover, Step 1 can be solved efficiently using existing algorithms such as PolytomySolver [5].

Having a binary refinement $\langle G,s_L\rangle$ of $\langle G^M,s^M_L\rangle$, the problem then reduces (Step 2) to finding a *b*-labeling for *G* allowing for an optimal DLE-Reconciliation.

Notice that, in contrast to the species labeling $s_L$, the *b*-labeling $b_L$ of the leaves of *G* is unknown after Step 1. The problem is therefore not reduced to extending a $b_L$ labeling to the internal nodes, but rather consists in finding an appropriate labeling $b_L$ of the leaves as well. This labeling is constrained by the *b*-labeling of $G^M$, as formulated in the next lemma which is directly deduced from the definition of a binary refinement (Definition 1).

#### Lemma 3

Let $\langle G^M,s^M_L,b^M_L\rangle$ be a multifurcated tree and $\langle G,s_L\rangle$ be a binary refinement of $\langle G^M,s^M_L\rangle$. Then $\langle G,s_L,b_L\rangle$ is a binary refinement of $\langle G^M,s^M_L,b^M_L\rangle$ if and only if, for any node *x* of *G* with a corresponding node (also denoted *x*) in $G^M$, $Count(L(\langle G^M,s^M_L,b^M_L\rangle [x])) = Count(L(\langle G,s_L,b_L\rangle [x]))$.

Therefore, in addition to $\langle G,s_L\rangle$ corresponding to a binary refinement of $\langle G^M,s^M_L\rangle$, the input of Step 2 also includes a set of constraints induced by the *b*-labeling of $V(G^M)$. These constraints can be represented as a set of $|\Sigma | \times 2$ matrices *M*(*x*) for each $x \in I$, where *I* is the subset of $V(G)\setminus L(G)$ with corresponding nodes in $V(G^M)$. The pair (*M*, *I*) is called the b-constraint of *G* (Fig. 2. (1)).

#### Definition 4

Given a binary tree $\langle G,s_L\rangle$ and a b-constraint labeling (*M*, *I*) for *G*, a labeling $b_L$ is said to be *consistent with (M, I)* if, for any $x\in I$, $Count(L(\langle G,s_L,b_L\rangle [x])= M(x)$.

Moreover, recall from Lemma 1 and Definition 3 that an optimal DLE-Reconciliation of a tree $\langle G,s_L,b_L\rangle$ is obtained from an optimal DL-Reconciliation of $\langle G,s_L\rangle$ by possibly converting duplication nodes to EGTs and adding a P/A labeling on edges. Moreover, as noted before, the *s* labeling of an optimal DLE-Reconciliation should be the LCA-Mapping. We denote it $s_{lca}$.

The main problem (Step 2) can thus be defined as follows. See an example in Fig. 2 where (1) is the input of the DLE-BinL problem and (2) is its output.

DLE-BinL Problem:

**Input:** A binary tree $\langle G,s_L\rangle$, a b-constraint (*M*, *I*) and a species tree *S*;

**Output:** An optimal DLE-Reconciliation $\langle G,s_{lca},b,e_V,e_E\rangle$ of $\langle G,s_L,b_L\rangle$ with *S*, where $b_L$ is a *b*-labeling consistent with (*M*, *I*).

Notice that, from Lemma 1, in the case of a unitary cost, the problem is equivalent to finding a minimum number of added EGTL events.

We call DLE-BinL1 the DLE-BinL problem where *I* is restricted to the root of *G* (which corresponds to considering a star-tree as the initial multifurcated tree).

## Complexity of the DLE-BinL and DLE-BinL1 problems

In this section, the considered cost is unitary; the complexity results are then naturally extendable to a general cost. The DLE-BinL problem in its decision version is defined bellow; the decision version of DLE-BinL1 is defined similarly.

DLE-BinL decision version:

**Input:** A binary tree $\langle G,s_L\rangle$, a b-Constraint (*M*, *I*), a species tree *S* and an integer *Cost*;

**Question:** Is there a DLE-Reconciliation $\langle G,s_{lca},b,e_V,e_E\rangle$ of $\langle G,s_L,b_L\rangle$ with *S* where $b_L$ is a *b*-labeling consistent with (*M*, *I*) for which $C(\langle G,s_{lca},b,e_V,e_E\rangle ) \le Cost$?

First observe that the DLE-BinL decision problem is in NP. In fact, given a DLE-Reconciliation $\langle G,s_{lca},b,e_V,e_E\rangle$ of $\langle G,s_L,b_L\rangle$, we can compute the cost of the DLE-Reconciliation (to verify if it is less than or equal to *Cost*) and verify if the *b*-labeling $b_L$ is consistent with (*M*, *I*) in polynomial time by traversing the tree *G*.

According to the considered Resolution method presented in "Problem statements" section, the input of Step 2 (finding an optimal DLE-Reconciliation of a binary gene tree) is not an arbitrary binary tree, but rather a binary refinement of an initial multifurcated tree $\langle G^M,s^M_L\rangle$, leading to an optimal DL-Reconciliation. In this section, we show that the DLE-BinL problem is NP-complete event with this requirement, in all but one very constrained version of the problem.

For a multifurcated tree $\langle G^M,s^M_L\rangle$, let ${\mathcal {B}}_{DL}(\langle G^M,s^M_L\rangle ,S)$ be the set of binary refinements of $\langle G^M,s^M_L\rangle$ leading to an optimal DL-Reconciliation with *S*. The DL-DLE-BinL (respec. DL-DLE-BinL1) decision problem is defined as the DLE-BinL (respec. DLE-BinL1) decision problem with the additional restriction that the binary tree given as input is in ${\mathcal {B}}_{DL}(\langle G^M,s^M_L\rangle ,S)$.

### Complexity of the DL-DLE-BinL1 problem

We first show, by reduction from Weighted Monotone one-in-three-satisfiability problem (Weighted Monotone 1-in-3-SAT Problem), that the DL-DLE-BinL1 decision problem is NP-complete. We can then deduce that DL-DLE-BinL is also NP-complete, as well as the more general DLE-BinL problem.

As the DLE-BinL decision problem is in NP, the DL-DLE-BinL1 decision problem is also in NP. The Weighted Monotone 1-in-3-SAT Problem is defined as follows (monotone meaning that there are no negation of variables in the clauses).

Weighted Monotone 1-in-3-SAT:

**Instance:** A set of clauses ${\mathcal {C}}= (C_1 \wedge C_2 \wedge \dots \wedge C_k)$ on a finite set ${\L} = \{\ell _1,\ell _2,\dots ,\ell _m\}$ of variables where each $C_i$, $1 \le i \le k$, is a clause of the form $(x \vee y \vee z)$ with $\{x,y,z\} \subseteq {\L}$ and a positive integer *n* ($n \le m$);

**Question:** Is there a truth assignment with exactly *n* variables set to True satisfying ${\mathcal {C}}$ such that exactly one literal in each clause is set to True?

As the Monotone 1-in-3-SAT problem is NP-complete, the Weighted Monotone 1-in-3-SAT problem is also NP-complete.

Given an instance ${\mathcal {I}}= ({\mathcal {C}}, {\L} ,n)$ of the Weighted Monotone 1-in-3-SAT problem, we compute, in polynomial time, a corresponding instance ${\mathcal {I}}' = (\langle G,s_L\rangle ,(M,I),S,Cost)$ of the DL-DLE-BinL1 decision problem.

First, the set of species $\Sigma$ is computed as follows:

- For each clause $C_i \in {\mathcal {C}}$, $1 \le i \le k$, $\Sigma$ contains the species $C_i$.
- For each clause $C_i \in {\mathcal {C}}$, $1 \le i \le k$ and for each $s \in \{1,\dots ,m-1 + (m-3)*k\}$, $\Sigma$ contains the species $T_{i_s}$.

Let $d=m-1 + (m-3)*k$. The species tree *S* is:
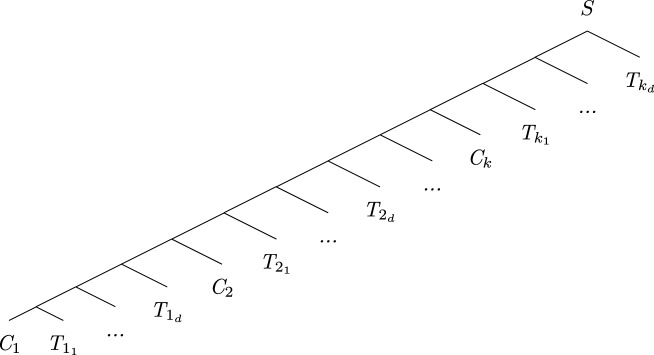


For $1 \le j \le m$, let $S_j$ be a gene tree species label isomorphic to *S* from which we removed all leaves $C_i$ ($1 \le i \le k$) such that $\ell _j$ is not in present the clause $C_i$.

The gene tree *G* is then:
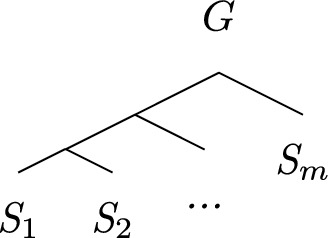


Notice that for each species $C_i$, $1 \le i \le k$, *G* contains exactly 3 leaves mapped to $C_i$ and that for each species $T_{i_s}$, $1 \le i \le k$, $1 \le s \le d$, *G* contains exactly *m* leaves mapped to $T_{i_s}$.

The b-constraint (*M*, *I*) is defined as follows:

- $$I=\{r(G)\}$$
- For each species $C_i$, $1 \le i \le k$, we require that one of the three leaves mapped to $C_i$ be labeled by 1 and that the remaining two leaves mapped to $C_i$ be labeled by 0.
- For each species $T_{i_s}$, $1 \le i \le k$, $1 \le s \le d$, we require that *n* of the *m* leaves mapped to $T_{i_s}$ be labeled by 1 and that the remaining $m - n$ leaves mapped to $T_{i_s}$ be labeled by 0.

Finally, *Cost* is set to *DL*(*G*, *S*).

#### Lemma 4

The gene tree $\langle G,s_L\rangle$ computed in the reduction is in ${\mathcal {B}}_{DL}(\langle G,s_L\rangle ,S)$.

#### Proof

Let $G^M$ be a star tree on the leaves of *G* and let ${\mathcal {R}}^*_{DL}$ be the optimal DL-Reconciliation of *G* with *S*. Notice that ${\mathcal {R}}^*_{DL}$ contains $m-1$ duplication nodes and $(m-3)*k$ losses and thus $C({\mathcal {R}}^*_{DL}) = m-1 + (m-3)*k$.

We will now show that for any binary refinement $G'$ of the star tree $G^M$, if the optimal reconciliation of $G'$ with *S* contains less than $(m-3)*k$ losses, then it contains at least $m-1 + (m-3)*k$ duplication nodes. Let ${\mathcal {R}}_{DL} = \langle R,s_{lca},e\rangle$ be the optimal DL-Reconciliation of $G'$ with *S*. Note that we consider here a non-compressed DL-Reconciliation. If the number of losses in ${\mathcal {R}}_{DL}$ is less than $(m-3)*k$, then there must exist *i* ($1 \le i \le k$) such that there are less than $m-3$ losses in the species in $\{C_i,T_{i_1},T_{i_2},\dots ,T_{i_d},p(C_i),p(T_{i_1}),p(T_{i_2}),\dots ,p(T_{i_d})\}$ in ${\mathcal {R}}_{DL}$. Let $\ell _0$ be the number of losses in $C_i$ in ${\mathcal {R}}_{DL}$ and let $\ell _s$ ($1 \le s \le d$) be the number of losses in $T_{i_s}$ in ${\mathcal {R}}_{DL}$. As exactly 3 leaves of $G'$ are mapped to $C_i$, there are $3+\ell _0$ non-duplication nodes of ${\mathcal {R}}_{DL}$ mapped to $C_i$. There is thus at most $3+\ell _0$ speciation nodes mapped to $p(C_i)$ in ${\mathcal {R}}_{DL}$ because a speciation node mapped to $p(C_i)$ must have one child mapped to $C_i$ (that child may be a duplication node mapped to $C_i$, but then this duplication node has at least two non-duplication nodes descendant mapped to $C_i$ that are not children of a speciation node mapped to $p(C_i)$). Using the same reasoning, there are at most $3+\ell _0+\ell _1$ speciation nodes mapped to $p(T_{i_1})$ in ${\mathcal {R}}_{DL}$. The same reasoning can be applied to show that for each node *x* in $\{p(T_{i_1}),p(T_{i_2}),\dots ,p(T_{i_d})\}$, there are less than *m* speciation nodes of ${\mathcal {R}}_{DL}$ mapped to *x* because $3+\sum _{s = 0}^{d}\ell _s < m$. For $1 \le s \le d$, as the *m* leaves of *R* mapped to $T_{i_s}$ cannot all have a speciation node as a parent, there is at least one duplication node mapped to $T_{i_s}$ in ${\mathcal {R}}_{DL}$. Therefore, there is at least $d=m-1 + (m-3)*k$ duplication nodes in ${\mathcal {R}}_{DL}$ and the cost of ${\mathcal {R}}_{DL}$ cannot be lower than the cost of ${\mathcal {R}}^*_{DL}$.

If otherwise, for a binary refinement $G'$ of the star tree $G^M$, the optimal reconciliation of $G'$ with *S* contains at least $(m-3)*k$ losses, then its cost is at least $m-1 + (m-3)*k$ because it contains at least $m-1$ duplication nodes as there are *m* leaves of $G'$ mapped to $T_{1_1}$. It thus cannot have a cost lower than $C({\mathcal {R}}^*_{DL})$.

We conclude that the gene tree $\langle G,s_L\rangle$ computed in the reduction is in ${\mathcal {B}}_{DL}(G,S)$. $\square$

We next show that ${\mathcal {I}}$ is a satisfiable instance of the Weighted Monotone 1-in-3-SAT problem if (Lemma 5) and only if (Lemma 6) its corresponding instance ${\mathcal {I}}'$ of the DL-DLE-BinL1 decision problem admits a DLE-Reconciliation of cost lower than or equal to *Cost*.

#### Lemma 5

Let ${\mathcal {I}}$ be a satisfiable instance of the Weighted Monotone 1-in-3-SAT problem. Then its corresponding instance ${\mathcal {I}}'$ of the DL-DLE-BinL1 decision problem admits a DLE-Reconciliation of cost lower than or equal to *Cost*.

#### Proof

Let ${\mathcal {R}}_{DL} = \langle G,s_{lca},e\rangle$ be the optimal DL-Reconciliation of *G* with *S*. We will show that we can obtain a DLE-Reconciliation ${\mathcal {R}}_{DLE}$ of cost lower than or equal to *Cost* from ${\mathcal {R}}_{DL}$ by converting some duplication events into EGT events. Recall that because the costs are unitary, converting a duplication event into an EGT event does not change the cost of the reconciliation.

Let *TA* be a truth assignment with exactly *n* variables set to True satisfying ${\mathcal {C}}$ such that exactly one literal in each clause is set to True (we know that such truth assignment exists because ${\mathcal {I}}$ is a satisfiable instance).

We now construct the *b*-labeling *b* (and $b_L$) and the mappings $e_V$ and $e_E$ as follows:

Let $e_V = e$. Let $e_E(x,y) = A$ for all edge (*x*, *y*) of *G*.

For all *j*, $1 \le j \le m$, such that $\ell _j$ is True (resp. False) in *TA*, we set $b(x) = 1$ (resp. $b(x) = 0$) for each node *x* of the subtree $S_j$. Let $j^*$ be the smallest index such that $\ell _{j^*}$ is set to False in *TA* (this index exists, as a truth assignment setting all variables to True cannot be a solution to the Weighted Monotone 1-in-3-SAT problem). If $j^* > 2$ we set $b(x) = 1$ for each node *x* on the path from the parent of $r(S_1)$ to the parent of $r(S_{j^*-1})$ and we set $b(y) = 0$ for each node *y* on the path from the parent of $r(S_{j^*})$ to *r*(*G*). Else (when $j^* \in \{1,2\}$), we set $b(x) = 0$ for each node *x* on the path from the parent of $r(S_1)$ to *r*(*G*).

There are no EGTL events in the subtrees $S_j$ ($1 \le j \le m$) because all nodes in a given subtree $S_j$ have the same *b*-label. Notice that all nodes on the the path from the parent of $r(S_1)$ to *r*(*G*) are duplication nodes in ${\mathcal {R}}_{DL}$ and we can convert them to EGT events in ${\mathcal {R}}_{DLE}$. If $j^* \in \{1,2\}$, then, for $1 \le j \le m$, if $\ell _{j}$ is set to True in *TA*, we set $e_V(\text {parent of } r(S_j)) = EGT$ (which is a transfer from 0 to 1). Else (when $j^* > 2$), then we set $e_V(\text {parent of } r(S_{j^*})) = EGT$ (which is a transfer from 0 to 1) and for $j^*+1 \le j \le m$, if $\ell _{j}$ is set to True in *TA*, we set $e_V(\text {parent of } r(S_j)) = EGT$ (which is a transfer from 0 to 1).

In both case, it is easy to see that this mapping is valid and that no EGTL events are required in ${\mathcal {R}}_{DLE}$.

As there are no EGTL events in ${\mathcal {R}}_{DLE}$, the cost of ${\mathcal {R}}_{DLE}$ is *DL*(*G*, *S*) and thus $C({\mathcal {R}}_{DLE}) \le Cost$.

For each leaf *x* of *G*, we set $b_L(x) = b(x)$. As exactly *n* variables are set to true in *TA* and as one variable per clause is set to True in *TA*, we know, by construction, that for each species $C_i$, $1 \le i \le k$, one of the three leaves mapped to $C_i$ is labeled by 1 and the remaining two leaves mapped to $C_i$ are labeled by 0 and that for each species $T_{i_s}$, $1 \le i \le k$, $1 \le s \le d$, *n* of the *m* leaves mapped to $T_{i_s}$ are labeled by 1 and the remaining $m - n$ leaves mapped to $T_{i_s}$ are labeled by 0. The *b*-labeling *b* we constructed is thus consistent with (*M*, *I*).

We then obtain a DLE-Reconciliation ${\mathcal {R}}_{DLE} = \langle G,s_{lca},b,e_V, e_E\rangle$ of $\langle G,s_L,b_L\rangle$ where $b_L$ is a *b*-labeling consistent with (*M*, *I*) for which $C({\mathcal {R}}_{DLE}) \le Cost$ and we conclude that the instance ${\mathcal {I}}'$ of the DL-DLE-BinL1 decision problem admits a DLE-Reconciliation of cost lower than or equal to *Cost*. $\square$

#### Lemma 6

Let ${\mathcal {I}}$ be an unsatisfiable instance of the Weighted Monotone 1-in-3-SAT problem. Then its corresponding instance ${\mathcal {I}}'$ of the DL-DLE-BinL1 decision problem does not admit a DLE-Reconciliation of cost equal or lower than *Cost*.

#### Proof

By contradiction, let us suppose that for an unsatisfiable instance ${\mathcal {I}}$ of the Weighted Monotone 1-in-3-SAT problem, its corresponding instance ${\mathcal {I}}'$ of the DL-DLE-BinL1 decision problem does admit an optimal DLE-Reconciliation ${\mathcal {R}}_{DLE}$ of cost equal or lower than *Cost*. In that case, ${\mathcal {R}}_{DLE}$ does not contain EGTL events as otherwise its cost would be greater than $DL(G,S) = Cost$ by Lemma 1.

As there are no duplication nodes in the DL-Reconciliation of the subtrees $S_j$ ($1 \le j \le m$) with *S*, we know from Lemma 1 that no EGT events occur in those subtrees in ${\mathcal {R}}_{DLE}$. Therefore, by definition of a DLE-Reconciliation, for $1 \le j \le m$, the nodes in $S_j$ have the same *b*-label.

We now define a truth assignment *TA* as follows: for all $1 \le j \le m$, set the variable $\ell _j$ to True if the *b*-label of the nodes in $S_j$ is 1, and set the variable $\ell _j$ to False otherwise.

For each species $C_i$ (corresponding to the clause $C_i$), $1 \le i \le k$, we know by construction that one of the three leaves mapped to $C_i$ is labeled by 1 and the remaining two leaves mapped to $C_i$ are labeled by 0 in *G*. Therefore the truth assignment *TA* satisfies ${\mathcal {C}}$ and for each clause $C_i$, one literal is set to True and two literals are set to False in *TA*. We know that exactly *n* variables are set to True in *TA*, as exactly *n* subtrees $S_i$ have their nodes labeled by 1 because of the b-constraint (*M*, *I*) requiring exactly *n* of the *m* leaves mapped to $T_{1_1}$ to be labeled by 1.

${\mathcal {I}}$ is then a satisfiable instance which is a contradiction. We thus conclude that if ${\mathcal {I}}$ is an unsatisfiable instance of the Weighted Monotone 1-in-3-SAT problem, then its corresponding instance ${\mathcal {I}}'$ of the DL-DLE-BinL1 decision problem does not admit a DLE-Reconciliation of cost equal or lower than *Cost*. $\square$

Since Weighted Monotone 1-in-3-SAT is NP-complete, Lemmas 5 and 6 lead to the following results.

#### Theorem 1

The DL-DLE-BinL1 decision problem is NP-complete.

#### Corollary 2

The DL-DLE-BinL and DLE-BinL decision problems are NP-complete.

### A tractable version of the DL-DLE-BinL1 problem

Given $\sigma \in \Sigma$, the *multiplicity* $M_{\langle G,s_L\rangle }(\sigma )$ of $\sigma$ in $\langle G,s_L\rangle$ is the cardinality of the set $\{x\in L(G) \;: \; s_L(x) = \sigma \}$. The *multiplicity factor* $M_{\langle G,s_L\rangle }$ is the constant defined as $\max _{\sigma \in \Sigma }M_{\langle G,s_L\rangle }(\sigma )$.

The two following lemmas make the link between the maximum number of non-loss nodes in an optimal DL-Reconciliation ${\mathcal {R}}_{DL}$ of $\langle G,s_L\rangle \in {\mathcal {B}}_{DL}(\langle G^M,s^M_L\rangle ,S)$ mapped to a given node in *S*, and the multiplicity factor $M_{\langle G,s_L\rangle }$. We will then show that the DL-DLE-BinL1 Problem is fixed parameter tractable with respect to the multiplicity factor $M_{\langle G,s_L\rangle }$.

#### Lemma 7

Let $G^M$ be a star-tree. For any optimal DL-Reconciliation ${\mathcal {R}}_{DL}$ of a tree $\langle G,s_L\rangle \in {\mathcal {B}}_{DL}(\langle G^M,s^M_L\rangle ,S)$, there are at most $M_{\langle G,s_L\rangle }$ speciation nodes of ${\mathcal {R}}_{DL}$ that are mapped to any given node in *S*.

#### Proof

We consider for this proof non-compressed reconciliations.

Let $k = M_{\langle G,s_L\rangle }$. Suppose there exists an optimal DL-Reconciliation ${\mathcal {R}}_{DL} = \langle R,s_{lca},e\rangle$ of a tree $\langle G,s_L\rangle \in {\mathcal {B}}_{DL}(\langle G^M,s^M_L\rangle ,S)$ for which, for a given node $\sigma$ in *V*(*S*), there are more than *k* speciation nodes of *R* that are mapped to $\sigma$. Let $x_1$, $x_2$,..., $x_{k+1}$ be any choice of $k+1$ speciation nodes among them. Note that the subtrees $R[x_1],R[x_2],\dots$, and $R[x_{k+1}]$ are *separated*, i.e. for any node *v* of *R*, *v* belongs to at most one of these subtrees.
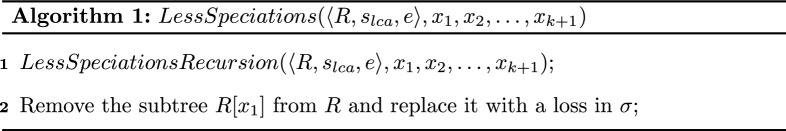

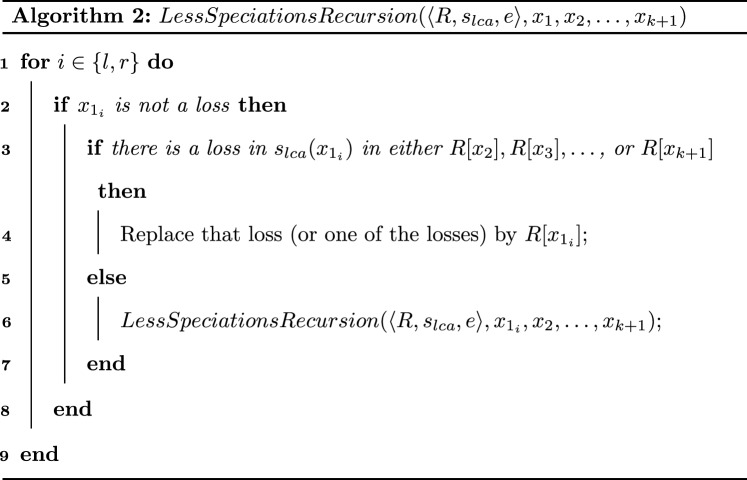


Consider Algorithm 1 above. We show that it transforms ${\mathcal {R}}_{DL}$ into another DL-Reconciliation ${\mathcal {R}}'_{DL}$ of another binary refinement of $G^M$ with one less speciation node mapped to $\sigma$ than ${\mathcal {R}}_{DL}$ and such that ${\mathcal {R}}'_{DL}$ has a lower cost than ${\mathcal {R}}_{DL}$. This contradicts the fact that ${\mathcal {R}}_{DL}$ is a reconciliation of a tree $\langle G,s_L\rangle$ belonging to ${\mathcal {B}}_{DL}(\langle G^M,s^M_L\rangle ,S)$.

It is straighfoward to see that this procedure leads to a valid DL-Reconciliation of a binary refinement of $G^M$ as all it does is replace the subtree $R[x_1]$ by a loss in $\sigma$ and place all the leaves belonging to $R[x_1]$ elsewhere in *R* in a position respecting definition 2 (because the procedure only replaces losses in *R* by subtrees of $R[x_1]$ which roots are mapped to the same species as the loss it replaces). In fact, every non-loss leaf of $R[x_1]$ belongs to a species which, by the hypothesis, cannot be the species label of more than $k-1$ other non-loss leaves of ${\mathcal {R}}_{DL}$, i.e. should be missing in at least one of the all separated subtrees $R[x_2],R[x_3],\dots R[x_{k+1}]$ of *R*.

This procedure never increases the number of duplication nodes in the reconciliation as it only replaces losses in *R* by subtrees of $R[x_1]$ whose root is mapped to the same species as the loss it replaces. It adds one new loss to the DL-Reconciliation as the subtree $R[x_1]$ is replaced by a loss in $\sigma$, and removes one loss every time a subtree of $R[x_1]$ replaces a loss in one of the subtrees $R[x_2],R[x_3],\dots$, or $R[x_{k+1}]$ (line 4 of Algorithm 2) and every time $x_{1_i}$ is a loss at line 2 of Algorithm 2. This happens at least twice: once for the left and once for the right subtree of $x_1$. Therefore in total, ${\mathcal {R}}'_{DL}$ has one less loss and no more duplications than ${\mathcal {R}}_{DL}$ and thus $C({\mathcal {R}}'_{DL}) < C({\mathcal {R}}_{DL})$. The result follows. $\square$

#### Lemma 8

Let $G^M$ be a star-tree. For any optimal DL-Reconciliation ${\mathcal {R}}_{DL}$ of a tree $\langle G,s_L\rangle \in {\mathcal {B}}_{DL}(\langle G^M,s^M_L\rangle ,S)$ with *S*, there are at most $2M_{\langle G,s_L\rangle }-1$ non-loss nodes of ${\mathcal {R}}_{DL}$ that are mapped to any given node in *S*.

#### Proof

As noted in the proof of Lemma 1, in an optimal DL-Reconciliation ${\mathcal {R}}$, a duplication node cannot have a loss as a child. It follows from that fact and from the definition of a DL-Reconciliation that for a given species $\sigma$ in $V(S)\setminus L(S)$ (respectively $\sigma \in L(S)$), the number of speciation nodes (respectively non-loss leaves) in ${\mathcal {R}}$ mapped to $\sigma$ is at least one more than the number of duplication nodes mapped to $\sigma$ and the number of non-loss leaves (respectively speciation nodes) mapped to $\sigma$ is 0. By Lemma 7, we know that for any optimal DL-Reconciliation ${\mathcal {R}}_{DL}$ of a tree $\langle G,s_L\rangle \in {\mathcal {B}}_{DL}(\langle G^M,s^M_L\rangle ,S)$ with *S*, the number of speciation nodes mapped to a given species is at most $M_{\langle G,s_L\rangle }$ (and, by definition, the number of non-loss leaves mapped to a given species is at most $M_{\langle G,s_L\rangle }$). Therefore the number of duplication nodes mapped to a given species is at most $M_{\langle G,s_L\rangle }-1$. Thus, there are at most $2M_{\langle G,s_L\rangle }-1$ non-loss nodes of ${\mathcal {R}}_{DL}$ that are mapped to any given node in *S*. $\square$

#### Lemma 9

Let $G^M$ be a star-tree. For any optimal DL-Reconciliation ${\mathcal {R}}_{DL}$ of a tree $\langle G,s_L\rangle \in {\mathcal {B}}_{DL}(\langle G^M,s^M_L\rangle ,S)$ with *S*, there are at most $3M_{\langle G,s_L\rangle }-1$ nodes of ${\mathcal {R}}_{DL}$ that are mapped to any given node in *S*.

#### Proof

From Lemma 8, for any optimal DL-Reconciliation ${\mathcal {R}}_{DL}$ of a tree $\langle G,s_L\rangle \in {\mathcal {B}}_{DL}(\langle G^M,s^M_L\rangle ,S)$ with *S*, the number of non-loss nodes mapped to a given species *x* is at most $2M_{\langle G,s_L\rangle }-1$. Moreover, in ${\mathcal {R}}_{DL}$, the parent of a loss node mapped to *x* is a speciation node mapped to *p*(*x*). By Lemma 7, we know that the number of speciation nodes mapped to *p*(*x*) is at most $M_{\langle G,s_L\rangle }$. Therefore, the number of nodes in ${\mathcal {R}}_{DL}$ mapped to *x* is at most $3M_{\langle G,s_L\rangle }-1$. $\square$

#### Lemma 10

Let ${\mathcal {R}}_{DL}=\langle R_{DL},s_{lca},e_{DL}\rangle$ be the optimal DL-Reconciliation (in its non-compressed form) of a gene tree $\langle G,s_L\rangle$ with a species tree *S* and $b_{DL}$ a *b*-labeling for the non-loss nodes of *R*. The optimal DLE-Reconciliation ${\mathcal {R}}_{DLE}=\langle R_{DLE},s_{lca},b_{DLE},e_{DLE}\rangle$ of $\langle G,s_L\rangle$ “consistent” with $b_{DL}$, i.e. with $b_{DLE}$ being an extension of $b_{DL}$, can be computed in *O*(*n*) time where $n = |L(G)|$.

#### Proof

We can do so by using Algorithm 1 in [2]. Note that in that paper, *EGTcopy* holds for an *EGT* event and *EGTcut* holds for an *EGTL* event. $\square$

Let ${\mathcal {R}}_{DL} = \langle R,s_{lca},e\rangle$ be a non-compressed DL-Reconciliation of a tree $\langle G,s_L\rangle$ with *S*. For the proof of the next Theorem, given a node $\sigma$ of *S*, we denote by $b[\sigma ]$ a given *b*-labeling for all non-loss nodes of *R* mapped to $\sigma$. Note that if there are *k* such nodes, then the number of possible $b[\sigma ]$ labelings is $2^k$. For a node $\sigma$ of *S*, we define $MaxTrees(\sigma )$ to be the set of “maximum” subtrees of *R* which roots are mapped to $\sigma$, i.e. such that the parent of these roots are not mapped to $\sigma$. For a node $\sigma \in V(S)\setminus L(S)$, we define $CutMaxTrees(\sigma )$ as the set of subtrees obtained from $MaxTrees(\sigma )$ by removing from the subtrees all strict descendants of the roots of the trees in $MaxTrees(\sigma _l)$ and $MaxTrees(\sigma _r)$. We also define, for any labeling $b[\sigma ]$, $CostMaxTrees(\sigma ,b[\sigma ])$ to be the sum of costs of the optimal DLE-Reconciliations consistent with $b[\sigma ]$ of all subtrees in $MaxTrees(\sigma )$. In addition, for any labelings $b[\sigma ]$, $b[\sigma _l]$ and $b[\sigma _r]$, $CostCutMaxTrees(\sigma ,b[\sigma ],b[\sigma _l],b[\sigma _r])$ is the sum of costs of the optimal DLE-Reconciliations consistent with $b[\sigma ]$, $b[\sigma _l]$ and $b[\sigma _r]$ of all subtrees in $CutMaxTrees(\sigma )$ with *S*.
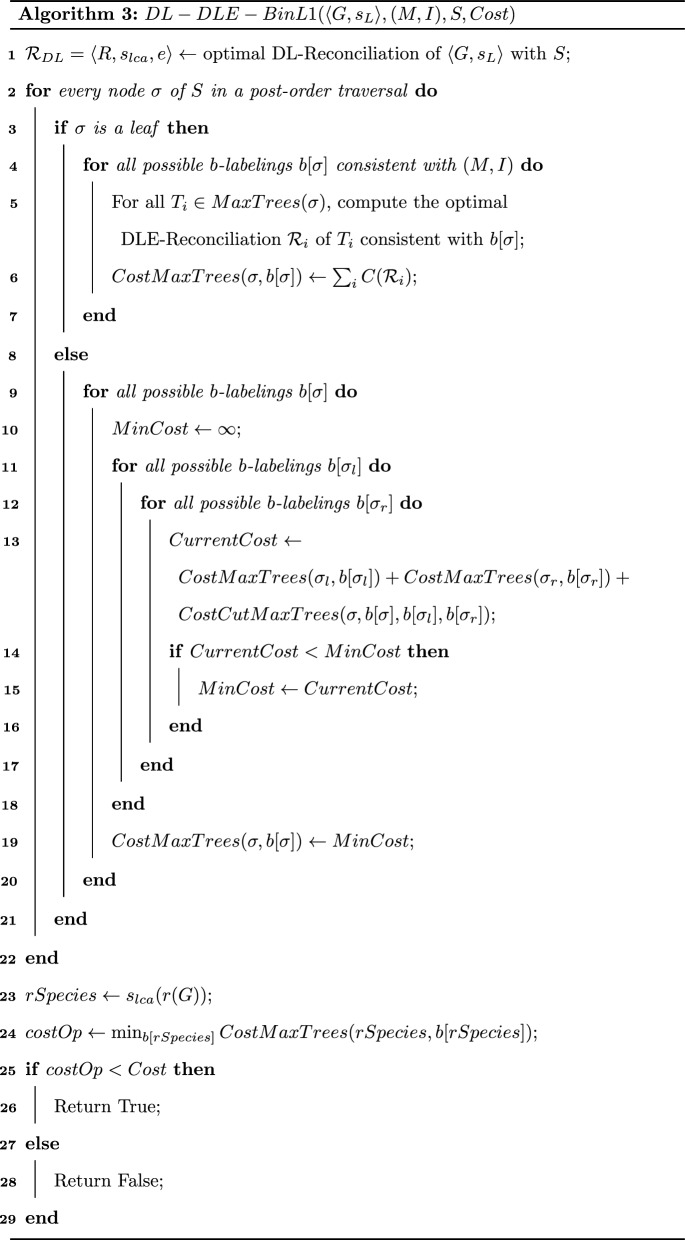


#### Theorem 2

The DL-DLE-BinL1 decision problem is fixed-parameter tractable with respect to the multiplicity factor $M_{\langle G,s_L\rangle }$.

#### Proof

Here, we consider non-compressed reconciliations.

We can solve the DL-DLE-BinL1 decision problem using Algorithm 3.

We show by induction that Algorithm 3 computes the correct cost $CostMaxTrees(\sigma ,b[\sigma ])$ for a given node $\sigma$ in *S* and all possible *b*-labelings $b[\sigma ]$.

If the node $\sigma$ is a leaf of *S*, then Algorithm 3 computes the correct $CostMaxTrees(\sigma ,b[\sigma ])$ by definition.

We may suppose now by the induction hypothesis that Algorithm 3 computes the correct cost for all possible *b*-labelings for the two children $\sigma _l$ and $\sigma _r$ of a given internal node $\sigma$ of *S*. Let show that Algorithm 3 is correct for $\sigma$. By the hypothesis, the algorithm correctly computes $CostMaxTrees(\sigma _l,b[\sigma _l])$ and $CostMaxTrees(\sigma _r,b[\sigma _r])$ for all possible $b[\sigma _l]$ and $b[\sigma _r]$. Note that in *R*, the two children of a non-loss and non-leaf node mapped to the node $\sigma$ are either mapped to $\sigma$ (if the node is a duplication) or to the children of $\sigma$ (if the node is a speciation). From that fact and by definition of a DLE-Reconciliation, for a given $b[\sigma ]$, $b[\sigma _l]$ and $b[\sigma _r]$,

$$CostMaxTrees(\sigma ,b[\sigma ]) = CostMaxTrees(\sigma _l,b[\sigma _l])+CostMaxTrees(\sigma _r,b[\sigma _r])+ CostCutMaxTrees(\sigma ,b[\sigma ],b[\sigma _l],b[\sigma _r])$$.

For a given $b[\sigma ]$, the algorithm tests all possibilities for $b[\sigma _l]$ and $b[\sigma _r]$ and thus the optimal one is found by the algorithm.

Note that, $MaxTrees(s_{lca}(r(G)) = \{R\}$. Thus, *costOp* computed in line 24 of Algorithm 3 is the cost of an optimal DLE-Reconciliation of $\langle G,s_L\rangle$ with *S*.

As for the complexity of the algorithm, from Lemma 8, we know that there are at most $2M_{\langle G,s_L\rangle }-1$ non-loss nodes of ${\mathcal {R}}_{DL}$ that are mapped to any given node $\sigma$ in *S*. The number of possible *b*-labelings for the nodes mapped to $\sigma$ is thus at most $2^{2M_{\langle G,s_L\rangle }-1}$. If $\sigma$ is a leaf of *S*, then all nodes of the subtrees in $MaxTrees(\sigma )$ are mapped to $\sigma$. Thus, for any *b*-labeling $b[\sigma ]$, the Lemma 10 applies and the optimal DLE-reconciliation consistent with $b[\sigma ]$ of each tree in $MaxTrees(\sigma )$ can be computed in linear time with its size. Moreover, the sum of the sizes of the subtrees in $MaxTrees(\sigma )$ is in $O(M_{\langle G,s_L\rangle })$ by Lemma 9. $CostMaxTrees(\sigma ,b[\sigma ])$ in line 6 can thus be computed in time $O(M_{\langle G,s_L\rangle })$. It follows that Lines 4 to 7 can thus be computed in time $O(M_{\langle G,s_L\rangle }2^{2M_{\langle G,s_L\rangle }})$.

Now for internal nodes, in line 13, $CostMaxTrees(\sigma _l,b[\sigma _l])$ and $CostMaxTrees(\sigma _l,b[\sigma _l])$ were previously computed and can be retrieved in constant time. Note that $b[\sigma ]$, $b[\sigma _l]$ and $b[\sigma _r]$ label all the nodes in $CutMaxTrees(\sigma )$. Thus, as shown previously, from Lemma 10 and Lemma 9, we deduce that $CostCutMaxTrees(\sigma ,b[\sigma ],b[\sigma _l],b[\sigma _r])$ can be computed in time $O(M_{\langle G,s_L\rangle })$. Thus, *CurrentCost* in line 13 can be computed in time $O(M_{\langle G,s_L\rangle })$. It follows that lines 9 to 20 can be computed in $O(M_{\langle G,s_L\rangle }8^{2M_{\langle G,s_L\rangle }})$.

The problem can thus be solved in time $O(n \times M_{\langle G,s_L\rangle }8^{2M_{\langle G,s_L\rangle }})$ where $n = |L(S)|$. $\square$

Finally, the next theorem states that, in contrast to DL-DLE-BinL1 and DL-DLE-BinL, the general problems DLE-BinL1 and DLE-BinL remain NP-complete even if the multiplicity factor of $\langle G,s_L\rangle$ is restricted to two.

#### Theorem 3

The DLE-BinL1 decision problem is NP-complete, even for $M_{\langle G,s_L\rangle }=2$.

The proof, given in Appendix, uses a reduction to the Monotone not-all-equal 3-satisfiability problem. The next corollary follows.

#### Corollary 3

The DLE-BinL decision problem is NP-complete, even for $M_{\langle G,s_L\rangle }=2$.

## The one-direction DLE-reconciliation problem

As endosymbiotic transfer events often move genes from the mitochondrial to the nuclear genome, and rarely in the opposite direction, we address the specific case where transfers are only allowed in one direction, i.e. when *b*-labels can only switch from 0 to 1, or only from 1 to 0. In the following definition, with no loss of generality, we assume transitions from 0 to 1.

### Definition 5

(One-direction DLE-Reconciliation) Let $\langle G,s_L,b_L\rangle$ be a rooted binary gene tree. A *One-direction DLE-Reconciliation* for $\langle G,s_L,b_L\rangle$ is a DLE-Reconciliation $\langle G,s_{lca},b,e_V, e_E\rangle$ verifying: for each edge (*x*, *y*) of *G*, if $b(x) \ne b(y)$ then $b(x) = 0$.

One-DLE-BinL Problem:

**Input:** A binary tree $\langle G_L,s_L\rangle$, a b-Constraint (*M*, *I*) and a species tree *S*;

**Output:** An optimal One-direction DLE-Reconciliation $\langle G,s_{lca},b,e_V, e_E\rangle$ of $\langle G,s_L,b_L\rangle$ with *S* where $b_L$ is a *b*-labeling consistent with (*M*, *I*).

We also define, in a similar way as before, the One-DLE-BinL1 problem where *I* is restricted to the root of *G*, and the corresponding decision problems. We next show that even this very restricted version of our initial problem is intractable. Moreover, the One-DL-DLE-BinL (respec.One-DL-DLE-BinL1) problem is defined as the One-DLE-BinL (respec. One-DLE-BinL1) problem with the additional restriction that the binary tree given as input is in ${\mathcal {B}}_{DL}(\langle G^M,s^M_L\rangle ,S)$.

We show that One-DL-DLE-BinL1 and One-DL-DLE-BinL are NP-hard but fixed parameter tractable with the multiplicity factor, while One-DLE-BinL1 and One-DLE-BinL are NP-hard even with a multiplicity factor of two.

### Theorem 4

The One-DL-DLE-BinL decision problem is NP-complete.

### Proof

The proof for NP-completeness of One-DL-DLE-BinL1 is the same as that of Theorem 1, as the DLE-Reconciliation in the proof verifies the One-direction condition. The NP-completeness of One-DL-DLE-BinL follows. $\square$

### Theorem 5

The One-DL-DLE-BinL1 is fixed parameter tractable with respect to the multiplicity factor $M_{\langle G,s_L\rangle }$.

### Proof

Note that the proof of Lemma 1 holds for a One-direction DLE-Reconciliation, i.e. an optimal One-direction DLE-Reconciliation can be obtained from the optimal DL-Reconciliation. Therefore, we can solve the One-DL-DLE-BinL1 Problem using the algorithm in the proof of Theorem 2, just giving an infinite cost for a transition from 1 to 0. $\square$

It follows from Theorem 4 that One-DLE-BinL is NP-complete. However, as for DLE-BinL1 and DLE-BinL, One-DLE-BinL1 and One-DLE-BinL remain NP-complete even if the multiplicity factor of $\langle G,s_L\rangle$ is restricted to two. The proof is given in Appendix.

### Theorem 6

The One-DLE-BinL1 and One-DLE-BinL decision problems are NP-complete, even for $M_{\langle G,s_L\rangle }=2$.

## A general algorithm for the DLE-BinL problem

A natural heuristic for the DLE-BinL problem for $\langle G,s_L\rangle$, where *G* is a binary resolution of an initial multifurcated tree with initial polytomies reflected by a b-Constraint (*M*, *I*), would be to solve each polytomy, i.e. each subtree rooted at a node *x* of *I*, individually, in a post-order traversal of the tree. In fact, this strategy leads to an exact algorithm for the DL Non-binary Reconciliation Problem [5]. However, in the case of DLE-Reconciliation, the *b*-labeling of internal nodes introduces a dependency between polytomies, avoiding the heuristic to be exact in general, i.e. for an arbitrary cost of operations. In this section, we present the general heuristic (Algorithm 4) and show that it is exact in the case of a unitary cost of operations.

Algorithm 4 traverses the tree *G* in post-order and each time it encounters a node $x \in I$, it “solves” the corresponding subtree *G*[*x*] and replaces it by a single leaf, with an appropriate *b*-label.

Once the tree *G* has been completely traversed, the subtrees are put back in the tree. Notice that on line 13, the algorithm adds a new intermediate species to $\Sigma$, but does not extend the species labeling $s_{lca}$ to this new species. The reason is that the new added species is eventually removed from the tree (line 25), i.e. does not remain in the returned reconciliation. Moreover, on line 9, the algorithm adds a new intermediate leaf without a *b*-label. Such nodes are technically ignored for the rest of the traversal of *G* and just used to re-graft the corresponding subtrees at the end (line 27).

Algorithm 4 calls a function $DLEBinL1(\langle G,s_L\rangle [x],M(x),S,Bin)$ where $Bin\in \{0,1\}$, returning an optimal solution of the DLE-BinL1 Problem such that $b(x) = Bin$. Recall that the DLE-BinL1 Problem is also NP-complete. In the next section, we will present *DLEBinL*1*OneSpecies* which can be substituted to *DLEBinL*1 in Algorithm 4 for a restriction of the problem, where, for each polytomy, genes belonging to the same species have the same *b*-label for all but one species.
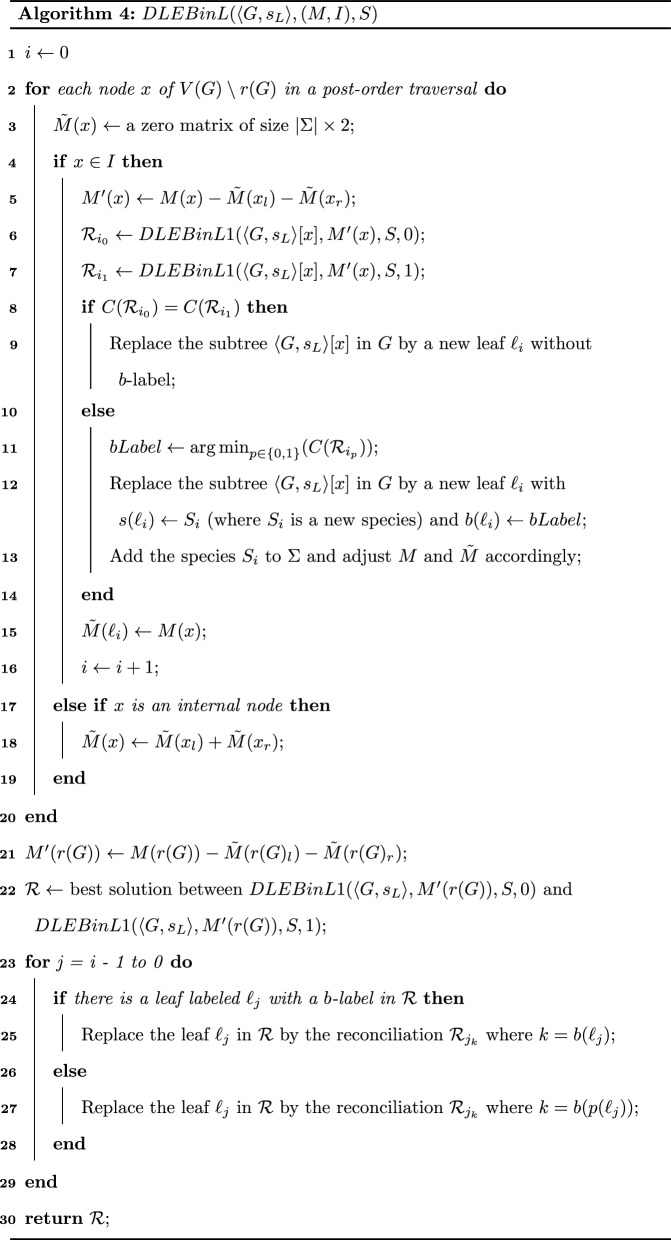


### Theorem 7

Let $\langle G,s_L\rangle$ be a binary tree, (*M*, *I*) be a b-Constraint for $\langle G,s_L\rangle$, *S* be a species tree. Then, with the input $(\langle G,s_L\rangle ,(M,I),S)$ and for a unitary cost, Algorithm 4 returns an optimal DLE-Reconciliation of $\langle G,s_L,b_L\rangle$ where $b_L$ is a *b*-labeling consistent with (*M*, *I*).

### Proof

The proof is by induction on the number of node $x \in V(G)$ such that $x \in I$.

Notice that the DLE-Reconciliation $\langle G,s_{lca},b,e_V,e_E\rangle$ returned by Algorithm 4 is such that *b* is a *b*-labeling consistent with (*M*, *I*) by construction.

If there is only one node $x \in V(G)$ such that $x \in I$, then this node *x* is the root of *G* by definition. The algorithm then returns an optimal solution, as we assume that we can solve $DLEBinLR(\langle G,s_L\rangle ,M'(r(G)),S,i)$ (where $M'(r(G)) = M(r(G))$) for $i \in \{0,1\}$.

If there is more than one node $x \in V(G)$ such that $x \in I$, then the root of *G* is in *I* by definition. By induction, we may assume that for each node $x \in V(G)\setminus r(G)$ such that $x \in I$, the reconciliation of *G*[*x*] computed by the algorithm is exact. For each of those subtrees *G*[*x*], we then know the possible *b*-label(s) at the root leading to an optimal reconciliation of *G*[*x*] and the corresponding optimal reconciliation of *G*[*x*]. We now give the index 1 to $|I| - 1$ to the elements of $I{\setminus } r(G)$. For all $1 \le j \le |I| - 1$, there is then two cases for $x_j \in I\setminus r(G)$:

1. $G[x_j]$ is such that both $b(x_j) = 0$ and $b(x_j) = 1$ can lead to an optimal reconciliation of $G[x_j]$. In that case, Algorithm 4 will remove $G[x_j]$ from *G* and replace it by a new leaf without a *b*-label. It solves $G(x_j)$ separately and then replace the new leaf in *G* by the solved $G[x_j]$ (after the rest of *G* is solved). $G[x_j]$ can be solved separately in that case, because regardless of the *b*-label of the parent of $G[x_j]$ in an optimal reconciliation of (the rest of) *G* we can obtain an optimal reconciliation of $G[x_j]$ with $r(G[x_j])$ having the same *b*-label as its parent (and thus we can obtain an optimal solution to the problem by putting the solved $G[x_j]$ with $r(G[x_j])$ having the same *b*-label as its parent back in *G*).
2. $G[x_j]$ is such that only $b(x_j) = i_j$ (where $i_j \in \{0,1\}$) can lead to an optimal reconciliation of $G[x_j]$. In that case, Algorithm 4 will remove $G[x_j]$ from *G* and replace it by a new leaf with *b*-label by $i_j$.

Then, Algorithm 4 solves $DLEBinLR(\langle G',s\rangle ,M'(r(G)),S,k)$ ($k \in \{0,1\}$) where $G'$ is the tree obtained after all the $x_j$ are visited by the algorithm. By construction, it will return the solution of lowest cost such that $b(x_j) = i_j$, for all $x_j$ belonging to Case 2.

Let’s show that this solution is optimal. By contradiction, suppose that there is $x_j \in I{\setminus } r(G)$ ($x_j$ belonging to Case 2) such that there is no optimal solution of the problem for which $b(x_j) = i_j$. Then, the optimal solution ${\mathcal {R}}^*$ of the problem is such that $b(x_j) \ne i_j$. In ${\mathcal {R}}^*$, if we set $b(x_j) = i_j$ and replace the reconciliation of the subtree $G[x_j]$ by the optimal reconciliation of $G[x_j]$ (that we can obtain because $b(x_j) = i_j$), we obtain a new solution ${\mathcal {R}}'$ of the problem with at most one more EGTL event (on the edge $(p(x_j),x_j)$) and such that the reconciliation of $G[x_j]$ in ${\mathcal {R}}'$ has a strictly lower cost than the reconciliation of $G[x_j]$ in ${\mathcal {R}}^*$. There is then at least one less event in the reconciliation of $G[x_j]$ in ${\mathcal {R}}'$ and as the cost are unitary, the solution ${\mathcal {R}}'$ is such that $C({\mathcal {R}}') \le C({\mathcal {R}}^*)$ and thus ${\mathcal {R}}'$ is optimal. Contradiction. We then conclude that there is an optimal solution of the problem for which $b(x_j) = i_j$.

Thus, Algorithm 4 returns an optimal solution for the input $(\langle G,s_L\rangle ,s_{lca},(M,I),S)$.

We conclude, by induction, that the solution returned by Algorithm 4 is optimal. $\square$

## An exact algorithm for the one-species version of the DLE-BinL1 problem

We consider a restriction of the DLE-BinL1 Problem where genes are specific to a single genome (the mitochondrial or nuclear genome) in all but one species. We call it the DLE-BinL1-OneSpecies problem. In its simplest version where a single species is present, the problem reduces to assigning a multiset of two labels (a given number of 0 s and a given number of 1 s) to the leaves of a tree-shape (i.e. a tree with no leaf labels), in a way minimizing 0–1 transitions in the tree. Similar problems on assigning leaves to tree-shapes or to multilabeled trees (MUL-trees) have been considered in the context of other tree distances (Robinson Foulds distance, path distance, maximum agreement subtree), most of them being NP-complete [12, 13]. Here, we present an exact polynomial-time algorithm for the DLE-BinL1-OneSpecies Problem.

Let $\sigma \in \Sigma$ be the only species for which the genes belonging to it are not specific to a single genome. We will call the leaves $\ell \in L(G)$ for which $s(\ell ) = \sigma$ *free leaves* and the leaves $\ell \in L(G)$ for which $s(\ell ) \ne \sigma$ *fixed leaves*. For a fixed leaf $\ell$, $b(\ell )$ is fixed and known in advance, as all leaves whose species label is $s(\ell )$ have the same *b*-label which is known from the matrix *M*. The DLE-BinL1-OneSpecies problem is then reduced to finding an optimal DLE-Reconciliation for which exactly *k* free leaves are labeled by 0, where $k = M(r(G))[\sigma ,0]$ (the $(\sigma ,0)$ entry of *M*(*r*(*G*))).

Let ${\mathcal {R}}_{DL} = \langle G,s_{lca},e\rangle$ be the optimal DL-Reconciliation for $\langle G,s_L\rangle$. From Lemma 1, any optimal DLE-Reconciliation ${\mathcal {R}}_{DLE} = \langle G,s_{lca},b,e_V,e_E\rangle$ with exactly *k* free leaves labeled by 0 can be obtained from ${\mathcal {R}}_{DL}$ by converting some duplications into EGTs and adding EGTL events, i.e. a *P*/*A* labeling on edges. We define $minCostTransfer(\langle G,s_{lca},b,e_V,e_E\rangle )= |e_{V_{EGT}}|* (\tau - \delta ) + |e_E|* \rho$. Then recall from "Preliminaries, evolutionary model and definitions" section that, by construction of ${\mathcal {R}}_{DLE}$, we have: $C({\mathcal {R}}_{DLE}) = DL(G,S) + minCostTransfer(\langle G,s_{lca},b,e_V,e_E\rangle )$.

The problem thus reduces to minimizing $minCostTransfer(\langle G,s_{lca},b,e_V,e_E\rangle )$.

We will need to consider the two possible *b*-labelings $i \in \{0,1\}$ for the root of *G*. We therefore denote by $minCostTransfer(\langle G,s_{lca},e\rangle ,i,k)$ the *minCostTransfer* function for an optimal DLE-Reconciliation ${\mathcal {R}}_{DLE}$ with exactly *k* free leaves labeled by 0 and with the additional constraint that $b(r(G)) = i$.

We are now ready to present Algorithm 5. It proceeds in two steps: (1) a bottom-up step (Algorithm 6) in which we assign an array of size $2 \times (k+1)$ to each node *x* of *G* where the (*i*, *j*)th entry equals $minCostTransfer(\langle G[x],s_{lca},e\rangle ,i,j)$; (2) a top-down step (not given in pseudo-code) in which the algorithm assigns the *b*-labeling of nodes and locates the EGT and EGTL events in the optimal solution. See Fig. 3 for an execution of Algorithm 5.
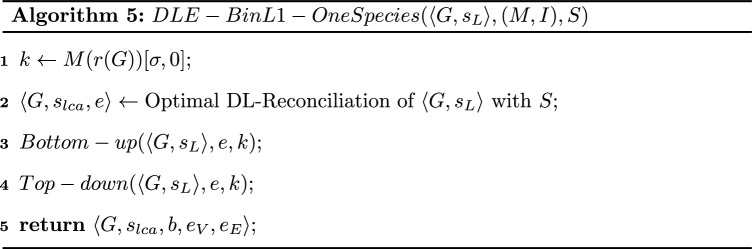

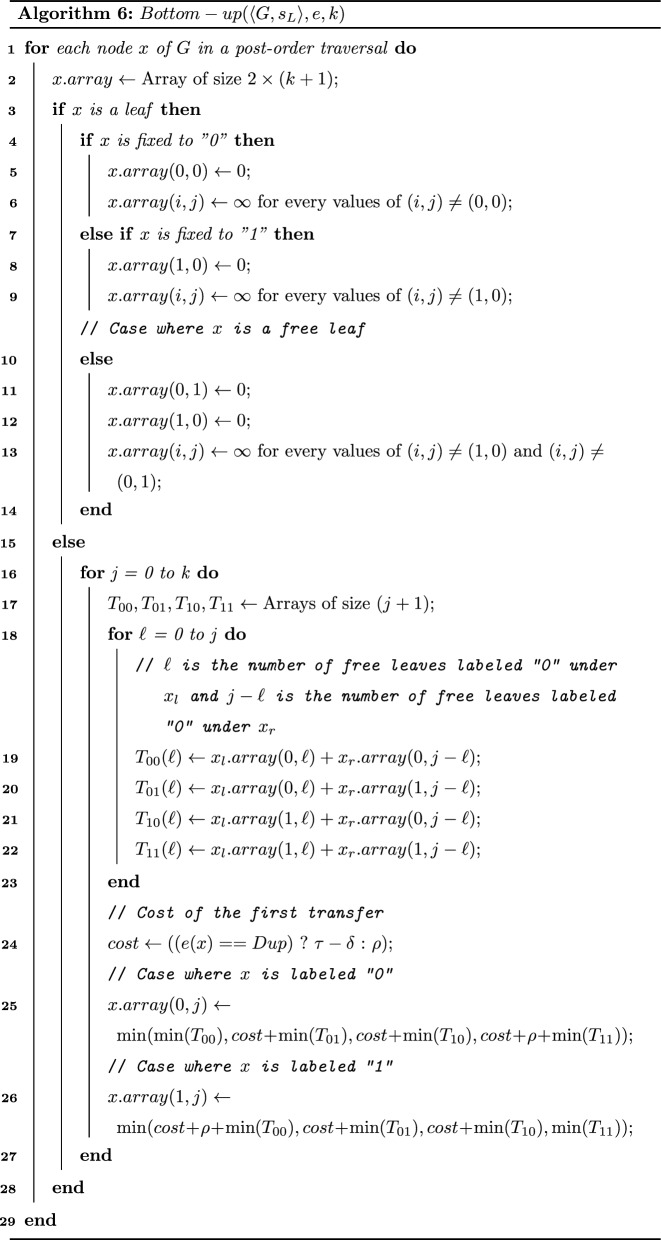


### Theorem 8

The output of Algorithm 5 is a solution of the DLE-BinL1-OneSpecies problem.

### Proof

Assume that, for each entry of *x*.*array* of each internal node *x*, Algorithm 6 keeps in memory pointers to the entries of the arrays of the children of *x* from which the value of the entry was obtained.

Once the optimal arrays are computed for all nodes, the optimal solution is easily reconstructed from the entry *min*(*r*(*G*).*array*(0, *k*), *r*(*G*).*array*(1, *k*)) by following the pointers from the root to the leaves.

The key point is therefore showing that the arrays computed by Algorithm 6 are exact, i.e., for each node *x*, *x*.*array*(*i*, *j*) is equal to $minCostTransfer(\langle G[x],s_{lca},e\rangle ,i,j)$ where $\langle G[x],s_{lca},e\rangle$ is the optimal DL-Reconciliation of *G*[*x*] with *S*. The proof is by induction.

If *x* is a leaf (either free or fixed), it is easy to see that *x*.*array* is correct.

Now, if *x* is an internal node, we may assume that $x_l.array$ and $x_r.array$ are correct by the induction hypothesis. By contradiction, let’s assume that there is (*i*, *j*) such that $x.array(i,j) \ne minCostTransfer(\langle G[x],s_{lca},e\rangle ,i,j)$. Let ${\mathcal {R}}$ be the optimal DLE-Reconciliation leading to $minCostTransfer(\langle G[x],s_{lca},e\rangle ,i,j)$. Then, in ${\mathcal {R}}$, $b(x) = i$, $b(x_l) = \ell _1$ where $\ell _1 \in \{0,1\}$ and $b(x_r) = \ell _2$ where $\ell _2 \in \{0,1\}$. Also, as there are *j* free leaves labeled by 0 under *x*, the sum of the numbers of free leaves labeled by 0 under $x_l$ and $x_r$ must be equal to *j*. If the genome labels of the children of *x* are not the same as *i*, *x* is converted as an EGT event if *x* is a duplication node in the DL-Reconciliation (and possibly an EGTL event is added) and if *x* is not a duplication node then some EGTL events may be added on the edges between *x* and its children. As the algorithm considers all possibilities of genome labels for $x_l$ and $x_r$ and all possibilities of number of free leaves labeled by 0 under $x_r$ and $x_l$ leading to *j* free leaves under *x* labeled to 0 (and considers the optimal assignation of EGT and EGTL events for the transfer(s) needed from *x* to its children), the particular possibility leading to ${\mathcal {R}}$ will be considered and then $x.array(i,j) = minCostTransfer(\langle G[x],s_{lca},e\rangle ,i,j)$. This is a Contradiction. Thus, there is no such (*i*, *j*) and *x*.*array* is exact.

We conclude, by induction, that the arrays computed by Algorithm 6 are exact. $\square$

### Theorem 9

Algorithm 5 computes the solution of the DLE-BinL1-OneSpecies problem in $O(nk^2)$ time, where $n = |L(G)|$.

### Proof

For each leaves of *G*, the associated array is computed in time *O*(*k*). For each internal node of *G*, the associated array is computed in time $O(k^2)$. The time complexity to compute the arrays for all the nodes is then $O(nk^2)$.

Once all the arrays are computed, the algorithm finds the optimal assignation of the internal nodes with a preorder traversal of *G* in time *O*(*n*)

We conclude that the time complexity of Algorithm 5 is $O(nk^2)$. $\square$

## Conclusion

In this paper, we present the first method for DLE-Reconciliation, that is a reconciliation accounting for duplications, losses, but also EGTs, for a multifurcated gene tree. It is a natural extension of the DL-Reconciliation of a multifurcated tree, where we first consider a solution for this problem, i.e. an optimal DL-Reconciliation, and then appropriately assign the binary *b*-labeling (0/1 for mitochondrial/nuclear) to the nodes of the tree in a way minimizing a total DLE (Duplications, Losses and EGTs) cost.

We show that the optimal *b*-labeling assignment step is NP-complete even if the gene tree in input is a binary refinement of a star-tree, and even when genes are present in only two copies in each species. Moreover, the problem remains NP-complete when the transfers are allowed in a single direction (e.g. only from 0 to 1) and even if the gene tree in input is an optimal resolution for the DL-Reconciliation. In this latter case, the problem is shown fixed-parameter tractable with respect to the gene tree’s multiplicity factor. We then present a greedy heuristic for the general version of the problem solving each polytomy independently in a bottom-up traversal of the tree. This heuristic is shown to be exact for a unitary cost of operations. Moreover, we give a polynomial-time algorithm for the resolution of a single polytomy in the case where genes are specific to a single genome in all but one species. We did not explore the case where genes are specific to a single genome in all but a fixed number of species, but we believe Algorithm 5 can be extended to solve this problem in polynomial time.

From a biological point of view, the next step will be to apply our method to the orthologous mitochondrial protein-coding genes (MitoCOGs) dataset [2, 10].

From a theoretical and algorithmic point of view, many open questions remain. Apart from the fact that a heuristic combining accuracy and time-efficiency should be developed for both the DLE-BinL and DLE-BinL1 problems in the general case, a more fundamental question is whether an exact one-step method, considering all the events at once, can be developed. In fact, the complexity results obtained here do not allow to conclude on the complexity of the DLE Non-binary Reconciliation problem. It is indeed not excluded that the polynomial-time PolytomySolver algorithm [5] can be extended for solving a multifurcated tree with a *b*-labeling of leaves, at least in special cases. In the near future, we will first explore the extension of PolytomySolver to the one species restriction of the model, before considering generalization to an arbitrary number of species.

## Data Availability

This declaration is not applicable.

## Appendix

### Proof of theorem 3

We show, by reduction from the Monotone not-all-equal 3-satisfiability problem (Monotone NAE3SAT Problem), that the DLE-BinL1 decision problem is NP-complete, even for $M_{\langle G,s_L\rangle }=2$.

Recall that the DLE-BinL1 decision problem is in NP as shown in "Complexity of the dle-binl and dle-binl1 Problems" section.

The Monotone NAE3SAT problem is the following (monotone meaning that there are no negation of variables in the clauses):

Monotone NAE3SAT:

**Instance:** A set of clauses ${\mathcal {C}}= (C_1 \wedge C_2 \wedge \dots \wedge C_k)$ on a finite set ${\L} = \{\ell _1,\ell _2,\dots ,\ell _m\}$ of variables where each $C_i$, $1 \le i \le k$, is a clause of the form $(x \vee y \vee z)$ with $\{x,y,z\} \subseteq {\L}$;

**Question:** Is there a truth assignment satisfying ${\mathcal {C}}$ such that the values in each clause are not all equal to each other?

Given an instance ${\mathcal {I}}= ({\mathcal {C}},{\L} )$ of the Monotone NAE3SAT problem, we compute, in polynomial time, a corresponding instance ${\mathcal {I}}' = (\langle G,s_L\rangle ,(M,I),S,Cost)$ of the DLE-BinL1 decision problem. First, the set of species $\Sigma$ is computed as follows:

- For $1 \le j \le m$, $\Sigma$ contains a species $\ell _j$ and for each clause $C_i \in {\mathcal {C}}$, $1 \le i \le k$ such that $\ell _j$ is in $C_i$, $\Sigma$ contains a species $\ell _{j_i}$.
- For each clause $C_i \in {\mathcal {C}}$, $1 \le i \le k$, $\Sigma$ contains the species $S_i{_1}$, $S_i{_2}$, $S_i{_3}$, $S_i{_4}$, $S_i{_5}$ and $S_i{_6}$.

For $1 \le j \le m$, let $L_j$ be a caterpillar tree on the leaves $\ell _{j_i}$ for all *i* such that $\ell _j$ is in the clause $C_i$. For $1 \le i \le k$, let $S_i$ be the tree computed as follows:

Then, the species tree *S* is:

Let now turn to defining the gene tree. For each clause $C_i = (x \vee y \vee z) \in {\mathcal {C}}$, $1 \le i \le k$, let $T_i$ be the following tree:

For $1 \le j \le m$, let $L'_j$ be a gene tree which is species label isomorphic to $L_j$. For $1 \le j \le m$, let $U_j$ be the tree computed as follows:

The gene tree *G* is then:

Notice that for each species $s \in \Sigma$, *G* contains exactly 2 leaves mapped to *s* and thus $M_{\langle G,s_L\rangle }=2$.

We set *M*(*r*(*G*)) equal to a matrix of ones of size $|\Sigma | \times 2$ (meaning that for each pair of leaves mapped to a given species *s*, we require one leaf to have a *b*-label 0 and the other to have a *b*-label 1). Also recall that $I=\{r(G)\}$. Finally, *Cost* is set to $DL(G,S) + k$.

We next show that ${\mathcal {I}}$ is a satisfiable instance of the Monotone NAE3SAT problem if (Lemma 12) and only if (Lemma 13) its corresponding instance ${\mathcal {I}}'$ of DLE-BinL1 decision problem admits a DLE-Reconciliation of cost lower than or equal to *Cost*.

#### Lemma 11

Let ${\mathcal {I}}$ be an instance of the Monotone NAE3SAT problem. For its corresponding instance ${\mathcal {I}}'$ of DLE-BinL1 decision problem, the optimal DLE-Reconciliation ${\mathcal {R}}_{DLE}$ is such that there is at least 1 EGTL event in each subtree $T_i$ of *G* (i.e. $e_E(x,y) = P$ for an edge (*x*, *y*) of $T_i$) for $1 \le i \le k$.

#### Proof

For the optimal DLE-Reconciliation ${\mathcal {R}}_{DLE}$, for each clause $C_i = (x \vee y \vee z) \in {\mathcal {C}}$, $1 \le i \le k$, for any *b*-labeling $b_L$ consistent with (*M*, *I*), there will be at least one EGTL event in the three following subtrees of $T_i$ (regardless of the labeling *b* of the internal nodes of these subtrees):

This is the case because there are no duplication node in the DL reconciliation of these subtrees with *S* (so no EGT events can occur in these subtrees in ${\mathcal {R}}_{DLE}$ by Lemma 1) and we know that at least one of these subtrees will not have all its leaves labeled by the same genome label (because two leaves with the same species label can’t have the same genome label by construction of the instance) so at least one EGTL will be required. $\square$

#### Lemma 12

Let ${\mathcal {I}}$ be an unsatisfiable instance of the Monotone NAE3SAT problem. Then its corresponding instance ${\mathcal {I}}'$ of DLE-BinL1 decision problem does not admit a DLE-Reconciliation of cost equal or lower than *Cost*.

#### Proof

By contradiction, let us suppose that for an unsatisfiable instance ${\mathcal {I}}$ of the Monotone NAE3SAT problem, its corresponding instance ${\mathcal {I}}'$ of the DLE-BinL1 decision problem does admit a DLE-Reconciliation of cost equal or lower than *Cost*. Let’s consider the optimal DLE-Reconciliation ${\mathcal {R}}_{DLE}$. ${\mathcal {R}}_{DLE}$ is optimal and thus $C({\mathcal {R}}_{DLE}) \le DL(G,S) + k$ as ${\mathcal {I}}'$ does admit a DLE-Reconciliation of cost equal or lower than $Cost = DL(G,S) + k$. By Lemma 11, ${\mathcal {R}}_{DLE}$ is such that there is at least 1 EGTL event in each subtree $T_i$ of *G* for $1 \le i \le k$. There is then at least *k* EGTL events in the reconciliation ${\mathcal {R}}_{DLE}$. As the cost of ${\mathcal {R}}_{DLE}$ is equal to *DL*(*G*, *S*) plus the number of EGTL events in ${\mathcal {R}}_{DLE}$ (from Lemma 4 in [2]), $C({\mathcal {R}}_{DLE})$ must be higher than or equal to $DL(G,S) + k$ and we conclude that $C({\mathcal {R}}_{DLE}) = DL(G,S) + k$. Thus, there is exactly one EGTL event in each subtree $T_i$ of *G* for $1 \le i \le k$ and no EGTL event elsewhere in the tree as otherwise $C({\mathcal {R}}_{DLE})$ would be higher than $DL(G,S) + k$. In particular, there is no EGTL event in the subtrees $U_j$, $1 \le j \le m$, and we can conclude that all nodes in the subtree $L_j'$, $1 \le j \le m$, have the same genome label (there is no EGT event in the subtree $L_j'$ as there is no duplication in the DL-Reconciliation of $L_j'$ with S).

We now define a truth assignment *TA* as follows: for all $1 \le j \le m$, set the variable $\ell _j$ to True if the genome label of the nodes in $L_j'$ is 1, and set the variable $\ell _j$ to False otherwise. We now show that *TA* satisfies ${\mathcal {I}}$. For each clause $C_i = (x \vee y \vee z) \in {\mathcal {C}}$, $1 \le i \le k$, we need to show that *x*, *y* and *z* are not all equal to each other. Let us suppose by contradiction that this is false, and that there exists a clause $C_i = (x \vee y \vee z) \in {\mathcal {C}}$ such that *x*, *y* and *z* are all equal to each other. Then, by construction, the genome labels of the leaves $x_i$, $y_i$ and $z_i$ in the corresponding subtrees $T_i$ are all equal to each other. Then, there is at least 2 EGTL events in $T_i$, as at least two of the following three subtrees of $T_i$ will not have all their leaves labeled by the same genome label and there are no EGT events in those subtrees (by construction) because there are no duplication node in the DL reconciliation of these subtrees with *S*:

This is a contradiction, as there must be exactly one EGTL event in the subtree $T_i$. We then conclude that for each clause $C_i = (x \vee y \vee z) \in {\mathcal {C}}$, $1 \le i \le k$, *x*, *y* and *z* are not all equal to each other. Thus, the truth assignment *TA* satisfies ${\mathcal {I}}$, and we conclude by contradiction that if ${\mathcal {I}}$ is an unsatisfiable instance of the Monotone NAE3SAT problem, then its corresponding instance ${\mathcal {I}}'$ of the DLE-BinL1 decision problem does not admit a DLE-Reconciliation of cost equal or lower than *Cost*. $\square$

#### Lemma 13

Let ${\mathcal {I}}$ be a satisfiable instance of the Monotone NAE3SAT problem. Then its corresponding instance ${\mathcal {I}}'$ of DLE-BinL1 decision problem admits a DLE-Reconciliation of cost lower than or equal to *Cost*.

#### Proof

Let ${\mathcal {R}}_{DL} = \langle G,s_{lca},e\rangle$ be the optimal DL-Reconciliation of *G* with *S*. We recall that, by definition, $C({\mathcal {R}}_{DL}) = DL(G,S)$. We will show that we can obtain a DLE-Reconciliation ${\mathcal {R}}_{DLE}$ of cost lower than or equal to *Cost* from ${\mathcal {R}}_{DL}$ by converting some duplication events into EGT events and by adding EGTL events. Notice that because the costs are unitary, converting a duplication event into an EGT event does not change the cost of the reconciliation. Thus, the cost of ${\mathcal {R}}_{DLE}$ is *DL*(*G*, *S*) plus the number of EGTL events in ${\mathcal {R}}_{DLE}$.

Let *TA* be a truth assignment satisfying ${\mathcal {C}}$ such that the values in each clause are not all equal to each other (we know that such truth assignment exists because ${\mathcal {I}}$ is a satisfiable instance).

We now construct the *b*-labeling *b* (and $b_L$) and the mappings $e_V$ and $e_E$ as follows:

Let $e_V = e$. Let $e_E(x,y) = A$ for all edge (*x*, *y*) of *G*.

For all *j*, $1 \le j \le m$, such that $\ell _j$ is True (resp. False) in *TA*, we set $b(x) = 0$ (resp. $b(x) = 1$) for each nodes *x* of the left subtree of $U_j$.

Notice that for each $\sigma \in \Sigma {\setminus } \{S_i{_j} | 1\le i \le k, 1 \le j \le 6\}$, we have set the genome label of exactly one of the two leaves of *G* for which the species label is $\sigma$. For each $\sigma \in \Sigma {\setminus } \{S_i{_j} | 1\le i \le k, 1 \le j \le 6\}$, we then set the genome label of the leaf with species label $\sigma$ whose genome label have not been set yet to $1 - i$ where *i* is the genome label of the other leaf with species label $\sigma$.

For each nodes *x* on the path from the parent of $r(T_1)$ to *r*(*G*), we set $b(x) = 0$. We set $b(r(T_i)) = 0$ for $1 \le i \le k$ and we set $b(r(U_j)) = 0$ for $1 \le j \le m$.

Therefore, there is no EGTL event on edges that are not in the subtrees $U_j$ ($1 \le j \le m$) or $T_i$ ($1 \le i \le k$), as all the nodes connected by those edges are labeled by 0.

We now show that no EGTL event is required in the subtree $U_j$ of *G*, for $1 \le j \le m$. By construction, all the nodes in the left subtree of $U_j$ have the same genome label *i* ($i \in \{0,1\}$) and the node in the right subtree of $U_j$ has the genome label $1 - i$. Thus, $b(r(U_j)_l)\ne b(r(U_j)_r)$. Notice that $r(U_j)$ is a duplication node in ${\mathcal {R}}_{DL}$ and recall that $b(r(U_j)) = 0$. We then set $e_V(r(U_j)) = EGT$ which is a transfer from 0 to 1. Therefore, there is no EGTL event in the subtree $U_j$.

We now show that exactly one EGTL event is required in the subtree $T_i$ of *G*, for $1 \le i \le k$. Notice that for any clause $C_i = (x \vee y \vee z) \in {\mathcal {C}}$, *x*, *y* and *z* can’t be all equal to each other in *TA* (because *TA* is a solution of the instance) and so, by construction, the genome labels of $x_i$, $y_i$ and $z_i$ in $T_i$ are not all equal to each other. Without loss of generality, let’s assume that $b(x_i) = 0$, $b(y_i) = 1$ and $b(z_i) = 0$ (the other possible cases are very similar). Then, the *b*-labeling of $T_i$ shown in Fig. 4 is correct and requires exactly one EGTL event.

We set $e_E(x,y) = P$ where (*x*, *y*) is the edge with a triangle on it in the tree above. We also set $e_V(lca_{T_i}(\{x_i,S_i{_4}\})) = EGT$, $e_V(lca_{T_i}(\{y_i,S_i{_5}\})) = EGT$ and $e_V(lca_{T_i}(\{z_i,S_i{_6}\})) = EGT$ (those are the nodes represented by a triangle in the tree above). We can do so because those nodes are duplication nodes in ${\mathcal {R}}_{DL}$.

There is then exactly *k* EGTL events in ${\mathcal {R}}_{DLE}$. Thus, the cost of ${\mathcal {R}}_{DLE}$ is $DL(G,S) + k$ and $C({\mathcal {R}}_{DLE}) \le Cost$.

For each leaf *x* of *G*, we set $b_L(x) = b(x)$. Notice that the *b*-labeling $b_L$ we constructed is consistent with (*M*, *I*) as for each $\sigma \in \Sigma$, there is one leaf labeled $\sigma$ whose genome label is 1 and one leaf labeled $\sigma$ whose genome label is 0, as required.

We then obtain a DLE-Reconciliation ${\mathcal {R}}_{DLE} = \langle G,s_{lca},b,e_V, e_E\rangle$ of $\langle G,s_L,b_L\rangle$ where $b_L$ is a *b*-labeling consistent with (*M*, *I*) for which $C({\mathcal {R}}_{DLE}) \le Cost$ and we conclude that the instance ${\mathcal {I}}'$ of the DLE-BinL1 decision problem admits a DLE-Reconciliation of cost lower than or equal to *Cost*. $\square$

Note that, by construction, the instance of the DLE-BinL1 decision problem in the reduction contains a gene tree with no more than two leaves having the same species label. From this remark, and since Monotone NAE3SAT is NP-complete, Lemmas 12 and 13 lead to the result.

### Proof of theorem 6

First observe that the One-DLE-BinL1 decision problem is in NP because the DLE-BinL1 decision Problem is in NP and because we can verify the one-direction condition in polynomial time.

We show that the One-DLE-BinL1 decision problem is NP-complete by reduction from the Monotone one-in-three 3-satisfiability problem (Monotone 1-in-3-SAT Problem) defined as follows (monotone meaning that there are no negation of variables in the clauses):

Monotone 1-in-3-SAT:

**Instance:** A set of clauses ${\mathcal {C}}= (C_1 \wedge C_2 \wedge \dots \wedge C_k)$ on a finite set ${\L} = \{\ell _1,\ell _2,\dots ,\ell _m\}$ of variables where each $C_i$, $1 \le i \le k$, is a clause of the form $(x \vee y \vee z)$ with $\{x,y,z\} \subseteq {\L}$;

**Question:** Is there a truth assignment satisfying ${\mathcal {C}}$ such that exactly one literal in each clause is set to True?

Given an instance ${\mathcal {I}}= ({\mathcal {C}},{\L} )$ of the Monotone 1-in-3-SAT problem, we compute, in polynomial time, a corresponding instance ${\mathcal {I}}' = (\langle G,s_L\rangle ,(M,I),S,Cost)$ of the One-DLE-BinL1 decision problem. The corresponding instance ${\mathcal {I}}'$ is the same as in the proof that the DLE-BinL1 decision problem is NP-complete (see"Complexity of the dle-binl and dle-binl1 Problems" section).

We next show that ${\mathcal {I}}$ is a satisfiable instance of the Monotone 1-in-3-SAT problem if (Lemma 14) and only if (Lemma 15) its corresponding instance ${\mathcal {I}}'$ of the One-DLE-BinL1 decision problem admits a DLE-Reconciliation of cost lower than or equal to *Cost*.

#### Lemma 14

Let ${\mathcal {I}}$ be an unsatisfiable instance of the Monotone 1-in-3-SAT problem. Then its corresponding instance ${\mathcal {I}}'$ of the One-DLE-BinL1 decision problem does not admit a DLE-Reconciliation of cost equal or lower than *Cost*.

#### Proof

The proof is similar to the proof of Lemma 12. All that is left to add to the proof for this restricted version is to show the following:

If for a given clause $C_i = (x \vee y \vee z) \in {\mathcal {C}}$ there are two of $b(x_i)$, $b(y_i)$, $b(z_i)$ equal to 1 and the other one equals to 0 (corresponding to a clause for which two variables are set to True and one variable is set to False), then the corresponding subtree $T_i$ will contain at least 2 EGTL events.

This is the case, as the only way to have only one EGTL event in the following subtrees of $T_i$ is to have an EGTL that transfers from 1 to 0, which is not allowed here (recall that there can be no EGT event in those subtrees because there are no duplication node in the DL-Reconciliation of these subtrees with *S*):

Then, ${\mathcal {I}}'$ cannot admit a DLE-Reconciliation of cost equal or lower than *Cost* if ${\mathcal {I}}$ is an unsatisfiable instance of the Monotone 1-in-3-SAT problem. $\square$

#### Lemma 15

Let ${\mathcal {I}}$ be a satisfiable instance of the Monotone 1-in-3-SAT problem. Then its corresponding instance ${\mathcal {I}}'$ of the One-DLE-BinL1 decision problem admits a DLE-Reconciliation of cost lower than or equal to *Cost*.

#### Proof

The proof is the same as the proof of Lemma 13. Indeed, if ${\mathcal {I}}$ is a satisfiable instance of the Monotone 1-in-3-SAT problem, then there is a truth assignment satisfying *C* such that the values in each clause are not all equal to each other (exactly one variable is set to True and two variables are set to False in each clause). In that case, for any clause $C_i = (x \vee y \vee z) \in {\mathcal {C}}$, one of $b(x_i)$, $b(y_i)$, $b(z_i)$ is equal to 1 and the other two are equal to 0. The proof of Lemma 13 then shows how to obtain a DLE-Reconciliation of cost lower than or equal to *Cost* verifying the One-direction transition condition. $\square$

Notice that, by construction, the instance of restricted the One-DLE-BinL1 decision problem in the reduction contains a gene tree with no more than two leaves having the same species label. From this remark, and since Monotone 1-in-3-SAT is NP-complete, Lemmas 14 and 15 lead to the result.
